# Detrimental and protective action of microglial extracellular vesicles on myelin lesions: astrocyte involvement in remyelination failure

**DOI:** 10.1007/s00401-019-02049-1

**Published:** 2019-07-30

**Authors:** Marta Lombardi, Roberta Parolisi, Federica Scaroni, Elisabetta Bonfanti, Alice Gualerzi, Martina Gabrielli, Nicole Kerlero de Rosbo, Antonio Uccelli, Paola Giussani, Paola Viani, Cecilia Garlanda, Maria P. Abbracchio, Linda Chaabane, Annalisa Buffo, Marta Fumagalli, Claudia Verderio

**Affiliations:** 1 IRCCS Humanitas, via Manzoni 56, 20089 Rozzano, Italy; 2 CNR Institute of Neuroscience, via Vanvitelli 32, 20129 Milan, Italy; 3 Department of Neuroscience Rita Levi-Montalcini and Neuroscience Institute Cavalieri Ottolenghi, University of Turin, Regione Gonzole 10, 10043 Orbassano, Italy; 4 Department of Excellence: Department of Pharmacological and Biomolecular Sciences (DiSFeB), University of Milan, via Balzaretti 9, 20133 Milan, Italy; 5 IRCCS Fondazione Don Carlo Gnocchi, via Capecelatro 66, 20148 Milan, Italy; 6 Department of Neurology, Rehabilitation, Ophthalmology, Genetics, Maternal and Child Health (DINOGMI), University of Genoa, Largo Paolo Daneo 3, 16132 Genoa, Italy; 7 IRCCS Ospedale Policlinico San Martino, Largo Rosanna Benzi 10, 16132 Genoa, Italy; 8 Department of Biotechnology and Translational Medicine, University of Milan, 20090 Segrate, Italy; 9 Humanitas University, via Manzoni 56, 20089 Rozzano, Italy; 10 Institute of Experimental Neurology (INSPE), San Raffaele Scientific Institute, Via Olgettina Milano 58, 20132 Milan, Italy

**Keywords:** Microglia, Extracellular vesicles, Mesenchymal stem cells, Myelin lesion, S1P, Astrocytes

## Abstract

Microglia are highly plastic immune cells which exist in a continuum of activation states. By shaping the function of oligodendrocyte precursor cells (OPCs), the brain cells which differentiate to myelin-forming cells, microglia participate in both myelin injury and remyelination during multiple sclerosis. However, the mode(s) of action of microglia in supporting or inhibiting myelin repair is still largely unclear. Here, we analysed the effects of extracellular vesicles (EVs) produced in vitro by either pro-inflammatory or pro-regenerative microglia on OPCs at demyelinated lesions caused by lysolecithin injection in the mouse corpus callosum. Immunolabelling for myelin proteins and electron microscopy showed that EVs released by pro-inflammatory microglia blocked remyelination, whereas EVs produced by microglia co-cultured with immunosuppressive mesenchymal stem cells promoted OPC recruitment and myelin repair. The molecular mechanisms responsible for the harmful and beneficial EV actions were dissected in primary OPC cultures. By exposing OPCs, cultured either alone or with astrocytes, to inflammatory EVs, we observed a blockade of OPC maturation only in the presence of astrocytes, implicating these cells in remyelination failure. Biochemical fractionation revealed that astrocytes may be converted into harmful cells by the inflammatory EV cargo, as indicated by immunohistochemical and qPCR analyses, whereas surface lipid components of EVs promote OPC migration and/or differentiation, linking EV lipids to myelin repair. Although the mechanisms through which the lipid species enhance OPC maturation still remain to be fully defined, we provide the first demonstration that vesicular sphingosine 1 phosphate stimulates OPC migration, the first fundamental step in myelin repair. From this study, microglial EVs emerge as multimodal and multitarget signalling mediators able to influence both OPCs and astrocytes around myelin lesions, which may be exploited to develop novel approaches for myelin repair not only in multiple sclerosis, but also in neurological and neuropsychiatric diseases characterized by demyelination.

**Electronic supplementary material:**

The online version of this article (10.1007/s00401-019-02049-1) contains supplementary material, which is available to authorized users.

## Introduction

Multiple sclerosis (MS) is a chronic demyelinating disease of the central nervous system characterized by secondary decline in myelin repair, loss of neurons and progressive disability [3, 28]. At early disease stages, successful remyelination is sustained by oligodendrocyte precursor cells (OPCs) [33]. In response to myelin damage, these cells, also called NG2-glia, proliferate and migrate to lesion sites, where they mature to new myelin-forming cells [64, 69] leading to restoration of rapid neurotransmission and neurological remission. During disease chronicization up to progressive MS, remyelination is gradually impaired, primarily not only due to the inability of OPCs to differentiate into myelinating cells, but also resulting from inefficient OPC activation and recruitment to myelin lesions [60]. At the cellular/molecular levels, the interplay between OPCs and microglia and other glial cells seems to play a key role in remyelination failure [54].

Microglia are the self-renewing, long-lived brain immune cells [35] whose tasks are mainly related to immune response and homeostasis. Guided by local environmental signals [57], microglia acquire a variety of activation states, ranging from protective [14, 72] to harmful [37] phenotypes participating in mechanisms of tissue repair as well as to injury. More than 80% of MS-specific genes identified at actively demyelinating lesions are related to microglia activation and T cell-mediated inflammation [66]. Moreover, interference with microglia activation limits disease progression [37], supporting a strong relationship between microglia activation and demyelination [28, 60]. Importantly, the negative impact of microglia on oligodendrocytes may occur through astrocytes, recognised players in MS immunopathology [12, 21], which are transformed to harmful A1 cells by inflammatory microglial mediators released from microglia (IL-1α, TNF and C1q) [55]. On the other hand, microglial gene expression patterns suggest favourable microglial function, such as phagocytosis of myelin debris and release of growth factors in acute lesions and at the active edges of chronic active lesions where remyelination often takes place [64, 69, 97] (reviewed in [18]) [57]. In addition, in the absence of pro-regenerative microglia, OPC differentiation becomes less efficient, eventually resulting in impaired myelin repair [64, 87], whereas a switch of microglia to an anti-inflammatory phenotype promotes remyelination and ameliorates the clinical signs of experimental autoimmune encephalomyelitis (EAE) [100], an animal model of MS. Despite the demonstration of both detrimental and beneficial effects of microglia on myelin lesion, the mode(s) of action of these cells in aiding or inhibiting remyelination is still largely unclear.

Extracellular vesicles (EVs), including ectosomes/microvesicles shed from the plasma membrane and exosomes originating from multivesicular bodies, have recently raised large interest as powerful mediators of intercellular communication between microglia and brain cells [71, 74, 90]. Production of microglial EVs dramatically increases under brain inflammation [22, 27, 61, 95] reflecting the extent of microglia activation. Following interaction with the recipient cells, microglia-derived EVs activate contact-mediated signaling pathways [1, 34] and/or deliver genetic information [25, 75, 95], thus profoundly influencing the molecular configuration and function of the target cells. Specifically, microglia-derived EVs propagate an activation signal to astrocytes and to other microglia [25, 95] and enhance excitatory neurotransmission [1]. However, whether microglial EVs play a role in the cross-talk between microglia and OPCs or not is still unknown.

Here, we have investigated the effects on OPCs of EVs released in vitro from either pro-inflammatory or pro-regenerative microglia both in vivo in the lysolecithin (LPC) mouse model of focal demyelination and in vitro in primary OPC cultures. Although classification in inflammatory and pro-regenerative cells underestimates the complexity of microglial activation, such a definition provides a useful framework for investigating the impact of microglia on OPCs. To induce pro-regenerative traits, microglia were exposed to the classical polarising agent IL-4 or to immunosuppressive mesenchymal stem cells (MSCs) [36, 101], while inflammatory state was obtained through stimulation with inflammatory cytokines.

We found that EVs produced by inflammatory microglia led to blockade of OPC differentiation to myelin-forming cells. Conversely, EVs released from microglia exposed to MSCs promoted remyelination. Biochemical fractionation combined with in vitro experiments revealed a major contribution of the inflammatory cargo of EVs to blocking OPC maturation, while surface lipids were identified as responsible factors for the pro-myelinating action of EVs. Although the type of lipids driving OPC differentiation still remains to be fully defined, sphingolipid sphingosine 1 phosphate (S1P) was unveiled as the key molecule favouring OPC migration, the first fundamental step in the remyelination process.

## Materials and methods

### Lysolecithin-induced myelin lesions

Demyelinating lesions were induced in the corpus callosum (CC) of adult (2.5–4 months old) and aged (8–12 months old) male C57BL/6 mice by stereotaxic unilateral injection of 1 μl of 2% lysolecithinto the mouse corpus callosum (CC, coordinates: 1.0 mm lateral, 1.0 mm rostral to Bregma and 1.5 mm deep). Three days post lesion (dpl), 2 × 10^8^ EVs, produced by 1.5 × 10^6^ microglia and dissolved in 150 µl of sterile saline, were delivered to the mice with mini-pumps (Alzet osmotic pumps 1007D) at the same coordinates over 4 days at 1.5 µl h^−1^ delivery rate. To limit EV degradation, minipumps were filled with freshly isolated EVs and implanted within a few hours. Controls were obtained with delivery of saline solution. To examine cell proliferation, we used the thymidine analog 5-bromo-2-deoxyuridine (BrdU), which is incorporated into DNA during S-phase of the cell cycle and remains in the DNA even when the cell has exited the active phases of the cell cycle. Mice were i.p. injected with BrdU (100 mg kg^−1^ body weight; 2 pulses 2 h apart) 1 day from the start of EVs delivery. A separate group of mice received a single injection of 6.6 × 10^7^ EVs, released from 0.5 × 10^6^ microglia and dissolved in 1 µl of sterile saline, at the site of lesion at 7 dpl. Controls were obtained by injecting saline. Mice were transcardially perfused with 4% paraformaldehyde in phosphate buffer (PB 0.1 M, pH 7.4) at 4 days after mini-pump implantation or at 3 days after acute injection and brains were post-fixed overnight and cryoprotected in 30% sucrose in 0.12 M PB. Surgery and perfusion were carried out under deep general anaesthesia (ketamine, 100 mg kg^−1^, Ketavet, Bayern, Leverkusen, Germany; xylazine, 5 mg kg^−1^, Rompun, Bayer, Leverkusen, Germany). All animal procedures were performed in accordance with the European directive (2010/63/EU) and the Italian Law for Care and Use of Experimental Animals (DL116/92; DL26/2014), and the studies were authorised by the Italian Ministry of Health (Authorization: 1112-2016PR) and the Bioethical Committee of the University of Turin.

### Electron microscopy of myelin lesions

Conventional electron microscopy was carried out as in [5]. LPC-lesioned old mice injected with saline, i-EVs or MSC-EVs were perfused transcardially with PB followed by 2% paraformaldehyde and 2.5% glutaraldehyde in PB. The brain was removed and post-fixed overnight at 4 °C in the same fixative. Vibratome sagittal sections (250 μm thick) were cut, and post-fixed with 1% osmium tetroxide for 1 h at 4 °C, then stained with uranyl acetate replacement stain (Electron Microscopy Sciences, USA). After dehydration in ethanol, samples were cleared in propylene oxide and embedded in Araldite (Fluka, Saint Louis, USA). Semithin sections (1 μm thick) were obtained at the ultramicrotome (Ultracut UCT, Leica, Wetzlar, Germany), stained with 1% toluidine blue and 2% borate in distilled water and then observed under a light microscope for precise lesion location. Ultrathin sections (70–100 nm) were examined under a transmission electron microscope (JEOL, JEM-1010, Tokyo, Japan) equipped with a Mega-View-III digital camera and a Soft-Imaging-System (SIS, Münster, Germany) for computerized acquisition of the images. Profiles of microglia, astrocytes, oligodendrocytes and myelinated axons were identified according to well established criteria [78]. Microglia were distinguished by their specific ultrastructural features including frequent stretches of endoplasmic reticulum and a condensed, electron-dense cytoplasm [7, 89]. In the core of the lesion, electron micrographs were taken at 25 K magnification and ImageJ software (https://imagej.nih.gov/ij/) was used to measure the proportion of myelinated axons (on at least 150 axons/animal) and perform the measurements needed to obtain G-ratios, the ratios of the axonal diameter (d white, Fig. 4k) to the outer fibre diameter including the myelin sheath (d yellow, Fig. 4k), of at least 50 myelinated axons/animal. Quantifications were performed on three mice (8–12 months old) per experimental condition.

### Immunohistochemistry

Brains were processed according to standard immunohistochemical procedures [9]. They were cut into 30 µm-thick coronal sections, collected in PBS and stained with the antibodies reported in the Table 1. OPCs were labelled by anti-NG2 antibodies while more mature cells were detected by anti-CC1 serum. Anti-Olig2 and anti-Sox10 stained the whole oligodendrocyte lineage. Anti-MBP antibodies labelled myelin. To follow cell proliferation, we used anti-BrdU. DAPI or Hoechst33258 were used to label nuclei. Anti-GFAP, anti-PTX3 and anti-C3 were used to label astrocytes, Iba-1 to reveal microglia. Sections were incubated overnight with primary antibodies at 4 °C in PBS with 1.5% donkey serum, 2% bovine serum albumin and 0.5% Triton X-100. Sections were then exposed for 2 h at room temperature to secondary anti-rabbit, anti-chicken antibodies conjugated with Alexa Fluor 488 or anti-rabbit antibodies conjugated with Alexa Fluor 546 (1:500; Invitrogen, Waltham, MA, USA), anti-mouse, anti rabbit antibodies conjugated with Cy3 (1:500; Jackson ImmunoResearch, West Grove, PA, USA), anti-goat antibodies conjugated with Alexa Fluor 649 (1:500; Invitrogen) or anti-mouse antibodies conjugated with Cy5 (1:500; Jackson ImmunoResearch). Double staining with rabbit anti-GFAP and rabbit anti-PTX3/anti-C3 was performed using the High Sensitivity Tyramide-Rhodaminate Signal Amplification kit (Perkin-Elmer, Monza, Italy) following the manufacturer’s instructions. Following counterstaining with 4,6-diamidino-2-phenylindole (DAPI) (Sigma-Aldrich Co., St. Louis, MO, USA) or Hoechst 33258 (Life Technologies, Monza, Italy) to label nuclei, slides were coverslipped with Mowiol (Millipore, Burlington, MA, USA) or with a fluorescent mounting medium from Dako (Milan, Italy). All images were collected using a Nikon Eclipse 90i confocal microscope (Nikon, Melville, NY, USA), or using a Zeiss LSM 800 confocal microscope (Carl Zeiss S.p.A, Milano, Italy). Adobe Photoshop 6.0 (Adobe Systems) was used to adjust image contrast and assemble the final plates. Measurements were derived from at least three sections per mice. Three to five animals were analysed per each time point or experimental condition. Image analysis was performed with the Image J software to quantify the proportion of NG2 or MBP-stained pixels throughout the entire lesioned area in each section [46, 53, 64]. Density of oligodendroglial, proliferative cells and PTX3- and C3-positive astrocytes in the lesion was calculated as number of cells mm^−2^, using the ImageJ software.

**Table 1:** List of primary antibodies used for immunohistochemistry (IHC), immunofluorescence (ICC) and western-blot (WB) analyses

Antibody	Host	Supplier	IHC dilution	ICC dilution	WB dilution
Anti-NG2	Rabbit	Millipore (Burlington, MA, USA)	1:200	1:100	
Anti-CC1	Mouse	Millipore (Burlington, MA, USA)	1:1500		
Anti-CNPase	Rabbit	SantaCruz (Dallas, Texas)			1:250
Anti-Olig2	Rabbit	Millipore (Burlington, MA, USA)	1:500	1:300	
Anti-SOX-10	Goat	SantaCruz (Dallas, Texas)	1:200		
Anti-MBP	Rat	AbD Serotec (Oxford, UK)		1:200	
Anti-MBP	Rat	Covance (Princeton, NJ)	1:1000		
Anti-MBP	Rat	Millipore (Burlington, MA, USA)		1:200	1:500
Anti-BrdU	Rat	Abcam (Cambridge, UK)	1:500		
Anti-pentraxin3	Rabbit	Home-made generated as previously described [10]	1:250		
Anti-C3	Rabbit	Dako (Milan, Italy)	1:250		
Anti-GFAP	Rabbit	Dako (Milan, Italy)	1:30,000 with amplification kit		
Anti-GFAP	Mouse	Sigma-Aldrich (Milan, Italy)		1:100	
Anti- Neurofilaments (SMI32)	Mouse	Cell Signaling (Beverly, MA, USA)		1:500	
Anti- Neurofilaments (SMI31)	Mouse	Cell Signaling (Beverly, MA, USA)		1:500	
Anti-α-tub	Mouse	Sigma-Aldrich			1:2000
Anti-GSTpi	Rabbit	MBL International Corporation (Sunnyvale, CA)			1:500
Anti-GPR17	Rabbit	Home-made generated as previously described, Ciana 2006			1:1000
Anti-GPR17	Rabbit	Cayman Chemical (Michigan, USA)		1:100	
Anti-GFP	Chicken	AVES Labs	1:700	1:1400	
Anti-GFP	Rabbit	Life Technologies (Monza, Italy)			1:3000
Anti-Iba1	Rabbit	Wako Chemicals (Richmond, VA)	1:1000		
4′,6-Diamidino-2-phenylindole DAPI		Fluka (Buche, Switzerland)	1:1000		
DAPI		Molecular Probes (Life Technologies, Monza, Italy)		1:20.000	
Hoechst 33258		Life Technologies (Monza, Italy)	1:50,000	1:10,000	
Anti-Alix	Rabbit	Covalab (Villeurbanne, France)			1:500
Anti-Flotilin 2	Mouse	BD transduction (San Jose, CA)			1:1000
Anti-GAPDH	Rabbit	Synaptic Systems (Goettingen, Germany)			1:1000
Anti-GS28	Mouse	BD transduction (San Jose, CA)			1:1000

### Magnetic resonance imaging (MRI)

In vivo MRI was performed using a 7 T scanner (Bruker-Biospin) equipped with a radiofrequency coil for mouse. Mice were anaesthetized with an intramuscular injection of Ketamine (100 mg kg^−1^; Ketavet, Bayern, Leverkusen, Germany) mixed with xylazine (5 mg kg^−1^; Rompun, Bayer, Leverkusen, Germany). High Resolution coronal and sagittal images, T2 weighted (TR/TE = 3000/48 ms, slice thickness = 0.7 mm, pixel = 78 × 80 µm) were acquired to localise lesions in the corpus callosum. Diffusion tensor imaging (DTI: 30 directions, *b* = 1000 s mm^−2^) was also used to measure water molecule diffusivity across white matter fibres to further characterise CC lesions.

### Primary cultures

Mixed glial cell cultures, containing both astrocytes and microglia, were established from rat Sprague–Dawley pups (P2) (Charles River, Lecco, Italy) and maintained for 10 days in the presence of South American foetal bovine serum (Life Technologies, Monza, Italy) that optimises microglia expansion or foetal bovine serum (EuroClone, Milan, Italy) which favours OPC proliferation.

#### Microglia

Microglia were harvested by orbital shaking for 40 min at 1300 r.p.m. and re-plated on poly-l-ornithine-coated tissue culture dishes (50 μg ml^−1^, Sigma-Aldrich, Milan, Italy). To minimise the activation, pure microglia (> 98%, [75] were kept for 24 h in low-serum (1%) medium. Cells were then stimulated with a cocktail of Th1 cytokines, i.e. 20 ng ml^−1^ IL-1β (Peprotech, Milan, Italy), 20 ng ml^−1^ TNF-α (Peprotech, Milan, Italy) and 25 ng ml^−1^ IFN-ɣ (Sigma Aldrich, Milan, Italy), or with 20 ng ml^−1^ IL-4 for 48 h (R&D, Milan, Italy). In addition, microglia were co-cultured in transwell with MSCs at a microglia-to-MSCs ratio of 1:1 for 48 h in the presence of Th1 cytokines. MSCs were plated on the filter of the upper chamber and microglia in the lower chamber of the transwell. At the end of treatment, MSCs were removed, microglia were washed and stimulated with ATP to increase EV production [75]. To obtain GFP-labelled EVs, microglia were established from GFP-transgenic rats expressing GFP under the chicken beta actin promoter [68]. Indeed, GFP is included in EVs released by GFP-positive microglia (Suppl. Figure 3h, Online resource).

#### OPCs

After microglia removal, OPCs growing on top of astrocyte monolayer were isolated by shaking cells on an orbital shaker at 200 rpm for 3 h and incubated on an uncoated Petri dish for 1 h to further eliminate microglia. Pure OPCs (> 95% [32] were seeded onto poly-d,l-ornithine-coated glass coverslips or plates (50 μg ml^−1^, Sigma-Aldrich, Milan, Italy) in Neurobasal (Life Technologies, Monza, Italy) supplemented with 2% B27 (Life Technologies, Monza, Italy), 2 mM l-glutamine (EuroClone, Milan, Italy), 10 ng ml^−1^ human platelet-derived growth factor BB (Sigma-Aldrich, Milan, Italy), and 10 ng ml^−1^ human basic fibroblast growth factor (Space Import Export, Milan, Italy), to promote proliferation (proliferating medium). After 3 days, cells were either detached with accutase (Millipore, Burlington, MA, USA) and used for migration assay, or switched to a Neurobasal medium lacking growth factors and supplemented with triiodothyronine T3 (10 ng ml^−1^, Sigma Aldrich, Milan, Italy) to allow differentiation (differentiating medium).

#### Dorsal root ganglion (DRG)-OPC co-cultures

DRG-OPC co-cultures were prepared according to a previously described protocol [31]. Briefly, DRG from E14.5 mouse embryos were plucked off from spinal cord, put in culture (1 DRG/coverslip) in Neurobasal supplemented with B27 in the presence of nerve growth factor (NGF) (100 ng ml^−1^; Harlan, Milan, Italy) and cycled with fluorodeoxyuridine (10 μM; Sigma Aldrich, Milan, Italy) to eliminate all non-neuronal cells. After 20 days, when neurites were well extended radially from DRG explants, 35 × 10^3^ OPCs were added to each DRG in culture and kept in Minimum essential media MEM (Life Technologies Monza, Italy) supplemented with glucose (4 g l^−1^; Sigma Aldrich, Milan, Italy), 10% FBS and 2 mM l-glutamine (EuroClone, Milan, Italy). Myelination was induced the following day by the addition of recombinant chimeric tyrosine kinase receptor TrkA Fc (1 μg ml^−1^; Sigma Aldrich, Milan, Italy) to the culture medium.

#### Astrocyte-OPC co-cultures

Purified astrocytes were isolated from P2 rat whole brains by magnetic-activated cell sorting (MACS) (Miltenyi Biotec, Bergisch Gladbach, Germany) with anti-GLAST (ACSA 1) MicroBeads according to the manufacturer’s instructions. After 1 week, OPCs were plated on top of astrocytes at an astrocyte-to-OPC ratio of 1:1. In a set of experiments astrocytes were isolated from P2 mouse whole brains and cultured alone for qPCR analysis of specific markers for A1 and A2 astrocytes.

#### MSCs

MSCs were prepared and expanded as described previously [102]. Briefly, marrow cells were flushed out from tibias and femurs of 6- to 8-week-old C57BL/6J mice and cultured in plastic plates as adherent cells using murine Mesencult as medium (Stem Cell Technologies, Vancouver, BC, Canada). Medium was refreshed every 3 days until cells reached 80% confluence. Following treatment with 0.05% trypsin solution containing 0.02% EDTA (Euroclone, Milan, Italy), the cells were plated in 75 cm^2^ flask at the density of 4 × 10^5^ cells. Mature MSCs, obtained after four to five passages in culture, were defined by the expression of CD9, Sca-1, CD73, and CD44 and the lack of the hematopoietic markers CD45, CD34, and CD11b on their surface, and their immunosuppressive activity was verified in T cell proliferation assays [102].

### RNA isolation and qRT-PCR

Total RNA was isolated from rat primary microglia using Direct-zol™ RNA MiniPrep (Zymo Research, Irvine, CA, USA) following the manufacturer’s protocol. cDNA synthesis was performed using High Capacity cDNA Reverse Transcription Kit (Applied Biosystems, Foster City, CA, USA) and Random Hexamers as primer. The resulting cDNAs were amplified using TaqMan^®^ Gene Expression Assay (Applied Biosystems, Foster City, CA, USA) using QuantStudio™5 (ThermoFisher Scientific, Waltham, MA, USA) real-time PCR system. The mRNA expression was normalised to the label of Rpl13 (Ribosomal Protein L13) mRNA. Data obtained were quantified using the 2^−ΔΔCT^ method [56]. Q-PCR for A1 and A2 markers was performed on murine astrocytes with the Luna Universal Probe qPCR Master Mix (M3004S, New England Biolabs) using the StepOne™ Plus Real-Time PCR System (Life Technologies, Monza, Italy). The expression of selected genes was normalized to the expression of the housekeeping gene β-actin. The list of primers used can be found in Table 2.

**Table 2:** List of gene expression assays for qPCR

Gene symbol	Name	Taqman assay
Arg 1	Arginase 1	Rn00691090_m1
C1q	Complement C1q	Rn01519903_m1
IL1-a	Interleukin 1 Alpha	Rn00566700_m1
IL1-β	Interleukin 1 Beta	Rn00580432_m1
iNOS	Nitric Oxide Synthase 2	Rn00561646_m1
Rpl13a	Ribosomal Protein L13	Rn00821946_g1
Socs-3	Suppressor Of Cytokine Signaling 3	Rn00585674_s1
TNF-α	Tumour Necrosis Factor	Rn99999017_m1
Amigo 2	Adhesion Molecule With Ig Like Domain 2	04688058001
β-Actin	Actin Beta	04688635001
CD14	Monocyte Differentiation Antigen CD14	04687574001
Ptx3	Pentraxin 3	04686993001
Tm4sf1	Transmembrane 4 L Six Family Member 1	04687990001
Serping1	Serpin Family G Member 1	04689011001

### EV isolation and quantification

EVs were isolated from microglia exposed to Th1 cytokine cocktail (i-EVs), IL-4 (IL4-EVs) or MSC (MSC-EVs). To isolate EVs, polarised microglia were stimulated with 1 mM ATP for 30 min in KRH (125 mM NaCl, 5 mM KCl, 1.2 mM MgSO_4_, 1.2 mM KH_2_PO, 2 mM CaCl_2_, 6 mM d-glucose, and 25 mM HEPES/NaOH, pH 7.4). The culture supernatant was collected and released EVs were pelleted at 100 k g after pre-clearing from cells and debris at 300 g, as previously described [34]. In some experiments, an ectosome-enriched fraction was pelleted at 10 k g. EV pellets were resuspended and used immediately after isolation. The number and dimension of EVs were assessed using NanoSight NS300 (NanoSight, UK) configured with a 488-nm laser and SCMOS camera. Videos were collected and analysed using the NTA-software (version 2.3), with the minimal expected particle size, minimum track length, and blue setting, all set to automatic. Camera shutter speed was fixed at 15 ms and camera gain was set to 300. Room temperature was ranging from 25 to 28 °C. EV pellets were re-suspended in 800 μl of 0.1 μm-filtered sterile KRH and four recordings of 30 s were performed for each sample.

EVs were destroyed by hypo-osmotic stress and re-pelleted to remove their luminal cargo as previously described [34]. For biochemical fractionation of EVs, total lipids were extracted with 2:1 (by volume) of chloroform and methanol. The lipid fraction was evaporated under a nitrogen stream, dried for 1 h at 50 °C and resuspended in PBS at 40 °C in order to obtain multilamellar vesicles. Small unilamellar vesicles were obtained by sonicating multilamellar vesicles, following the procedure of [4].

### Quantification of sphingolipid content in EVs

[^3^H] Sphingosine ([^3^H]Sph) was stocked in absolute ethanol. For the quantification of sphingolipid content in EVs, 1 × 10^6^ microglia were pulsed with [^3^H]Sph (0.3 µCi ml^−1^) for 24 h followed by a 48-h chase in order to obtain a [^3^H]GM3/[^3^H]SM ratio corresponding to that of endogenous compounds (data not shown), an index for a steady-state labelling of cell sphingolipids [96]. At the end of the chase, microglia were stimulated with ATP, EVs were pelleted and the cells were washed twice with PBS at 4 °C and harvested. Total lipids were extracted from both EVs and cells and processed as previously described [79, 80]. The organic phase and the aqueous phase were analysed by high-performance thin-layer chromatography (HPTLC) using chloroform/methanol/water (55:20:3 by volume) and butanol/acetic acid/water (3:1:1 by volume) as the solvent systems.

### Raman spectroscopy

The Raman analysis was performed following a previously described protocol [40]. Briefly, freshly isolated EVs were laid on a calcium fluoride slide and allowed to air dry. All of the measurements were performed with a Raman microspectroscope (LabRAM Aramis, Horiba Jobin–Yvon S.A.S, Lille, France) equipped with a laser line operating at 532 nm and a Peltier-cooled CCD detector. Acquisitions were performed with 50X objective (NA 0.75, Olympus, Tokyo, Japan), 1800 grooves mm^−1^ diffraction grating, 400 μm entrance slit and confocal mode (600 μm pinhole) in the spectral ranges 600–1800 cm^−1^ and 2600–3200 cm^−1^. Accumulation times were 2 × 30 s per spectrum. Silicon reference sample was used for the instrument calibration. At least 30 independent replicates of the Raman spectra were obtained for every EV type. After acquisition, polynomial baseline correction and unit vector normalisation were performed before the multivariate statistical analysis.

### OPC migration assay

Migration of OPCs was performed in Boyden chambers (8 μm pore size filter; Constar, Corning, NY, USA) as previously described [11]. Briefly, the chamber was nested inside the well of 24-well plates and 5 × 10^4^ OPCs were seeded in the top of each insert with 200 μl of neurobasal medium. The bottom well was filled with 600 μl of medium containing EVs released from 1 × 10^5^ microglia (~ 4 × 10^8^ EVs ml^−1^). The chemotactic agent sphingosine-1 phosphate (S1P) (100 nM) was used as positive control. After 16 h, non-migrated cells were removed from the top compartment with a cotton swab, whereas cells that had migrated to the lower side of the filter were fixed with 4% paraformaldehyde and stained with Hoechst33258 (Life Technologies, Monza, Italy). Images were acquired at 20× magnification under an inverted fluorescence microscope (200 M; Zeiss, Oberkochen, Germany) and cells were counted using ImageJ cell counter plugin in 45 random fields per well. All conditions were run in triplicate. Data are expressed as a percentage of basal migration that is the migration of OPCs without chemoattractant.

### EV delivery by optical manipulation

An IR laser beam (1064 nm, CW) for trapping was coupled into the optical path of an inverted microscope (Axiovert 200 M, Zeiss, Oberkochen, Germany) through the right port of the microscope. The trapping beam was directed to the microscope lens (Zeiss 63X, NA 1.4) by the corresponding port mirror (100%) and the tube lens. Optical trapping and manipulation of EVs were performed following the approach previously described [73]. Immediately before recording, EVs were added in the temperature-controlled recording chamber, where OPCs plated on glass coverslips were maintained in 400 μl of Neurobasal medium with B27. As soon as an EV appeared in the recording field, it was trapped and positioned on a selected OPC by moving the cell stage horizontally and the microscope lens axially. After about 30 s from initial contact, the laser was switched off to prove EV-OPC interaction, as previously described [73]. During the experiments, OPCs were live-imaged with a spinning disk confocal microscope (UltraVIEW acquisition system, Perkin Elmer Waltham, MA, USA) using a digital camera (High Sensitivity USB 3.0 CMOS Camera 1280 × 1024 Global Shutter Monochrome Sensor, Thorlabs, Newton, NJ, USA) at a frame rate of 2 Hz.

### OPC proliferation assay

One day after plating on glass coverslips, OPCs were co-exposed to EVs derived from twice as many microglia (~ 2 × 10^7^ EVs/500 µl) and to the thymidine analog EdU (Click-iT^®^ EdU Assay, Life Technologies, Monza, Italy) for 24 h in proliferating medium. Cells were fixed with 4% paraformaldehyde and stained for EdU following the manufacturer’s instructions. Coverslips were then incubated with DAPI (1:20000, Molecular Probes, Life Technologies) to reveal total nuclei and mounted with a fluorescent mounting medium (Dako, Milan, Italy). 40–50 fields per coverslip were imaged at 20× magnification under an inverted fluorescence microscope (200 M Zeiss, Oberkochen, Germany) connected to a PC computer equipped with the Axiovision software (Zeiss). OPC proliferation was assessed by quantifying EdU-DAPI double positive nuclei in at least three coverslips/condition, using ImageJ cell counter plugin. Cells with nuclei larger than 15 μm, belonging to astrocytes occasionally present in the cultures, were excluded from the analysis.

### OPC differentiation assay

OPCs were kept for 3 days in proliferating medium, shifted to differentiating medium for 24 h and then exposed to EVs for 48 h (2 × 10^7^ EVs/500 µl). Cells were fixed and labelled with anti-G Protein-Coupled Receptor 17 (GPR17) and anti-MBP (Table 1) in Goat Serum Dilution Buffer (GSDB; 450 mM NaCl, 20 mM sodium phosphate buffer, pH 7.4, 15% goat serum, 0.3% Triton X-100), followed by secondary antibodies conjugated to Alexa Fluor 555 or Alexa Fluor 488 (1:200; Molecular Probes, Life Technologies). Differentiation towards mature oligodendrocytes was determined by counting MBP^+^ cells over total DAPI+ cells in 35–45 fields per coverslip with ImageJ software. GPR17 staining was used to reveal immature oligodendrocytes, the most abundant oligodendrocyte population after 3 day in differentiating medium.

### OPC myelination assay

OPC-DRG co-cultures were kept in 1 μg ml^−1^ TrkA-Fc for 5 days and then exposed to EVs for 11 days (fresh EVs, 2 × 10^7^ EVs/500 µl, were added at day in vitro (DIV5, DIV8 and DIV12). Cells were fixed at DIV16 with paraformaldehyde and labelled with anti-MBP and anti-high-molecular-weight neurofilaments (NF) antibodies (SMI31 and SMI32 in Table 1) in GSDB, followed by secondary antibodies conjugated to Alexa Fluor 555 or Alexa Fluor 488 (1:600; Molecular Probes, Life Technologies). Nuclei were labelled with DAPI. Coverslips were mounted with a fluorescent mounting medium (Dako, Milan, Italy). For the co-culture analysis, stacks of images of MBP- and SMI31- and SMI32-positive cells were taken under confocal microscope at 40× magnification (at 6 fields/coverslip) and the ZEISS LSM Image Browser was utilised to automate quantification of the myelination index. Images in the stack were merged at each level and pixels overlapping in the red and green fields above a predefined threshold intensity value were highlighted in white. The amount of myelin per axon (myelination index) was calculated as the ratio between the white pixel and the green pixel areas.

### Western blot analysis

OPCs were lysed with a buffer containing 1% sodium dodecyl sulphate (SDS), 10 mM HEPES, 2 mM EDTA pH 7.4. A modified version of the Laemmli buffer (20 mM Tris pH 6.8, 2 mM EDTA, 2% SDS, 10% glycerol, 2% β-mercaptoethanol, 0.01% bromophenol blue) was then added to a final 1× concentration and proteins were separated by electrophoresis, blotted on nitrocellulose membrane filters and probed using the using the primary antibodies reported in the Table 1 and the HRP-conjugated secondary antibodies (goat anti-rat 1:1000, goat anti-rabbit 1:4000 and goat anti-mouse 1:2000; Sigma Aldrich, Milan, Italy). Photographic development was by chemiluminescence (ECL, GE Healthcare) according to the manufacturer’s instructions. Densitometric analysis of the protein bands was performed with ImageJ software. Band intensities were measured as integrated density volumes (IDV) and expressed as percentage of control lane values.

### ELISA

The concentration of IL-1a or C1q or TNF-α in microglial cells or EVs was quantified using a solid-phase sandwich ELISA (enzyme-linked immunosorbent assay) kit following the manufacturer’s protocol (Rat IL-1a ELISA kit, Invitrogen Waltham, MA, USA, BMS627; Rat TNF-α ELISA kit, Invitrogen Waltham, MA, USA, BMS622, Rat C1q ELISA kit, Novus Biologicals Centennial, CO, USA, NBP2-74988). IL-1a or C1q or TNF-α content in the cells was determined after cell lysis with RIPA Buffer (Sigma Aldrich, Milan, Italy), whereas inside EVs after detergent permeabilization with 0.6% Triton X-100 (Sigma Aldrich, Milan, Italy) in the presence of protease inhibitors (1:1000, Sigma Aldrich, Milan, Italy). Sample absorbance was measured with a spectrophotometric system (1420 Multilabel Counter Victor 2; Wallac) at 450 nm at 10 Hz. The amount of IL-1a, C1q or TNF-α in EVs was estimated on the basis of a standard curve in the presence 0.6% Triton X-100.

### Cryo-electron microscopy

Cryo-EM allows imaging of samples without the addition of any heavy metals or fixatives, which might cause artefacts, with the drawback of yielding a lower contrast. The sample is frozen so rapidly that the water vitrifies forming no ordered crystals, and the native structure of the sample is preserved [26, 70]. Freshly prepared vesicles resuspended in saline were plunge frozen in liquid propane using a Vitrobot Mark IV (ThermoFisher Scientific, Oregon, USA).

### Drugs and reagents

S-FTY720-Vinylphosphonate (kind gift from Prof. Robert Bittman) was dissolved in fatty acid-free bovine serum albumin (1 mM in PBS). S1P (Enzo Biochem. Inc, Farmingdale, NY, USA) was dissolved in fatty acid-free bovine serum albumin (4 mg ml^−1^ in distilled water). Stock solutions were diluted in fresh Krebs–Ringer solution.

### Statistical analysis

All data are presented as mean ± SEM from the indicated number of experiments “N”. Statistical analysis was performed using SigmaStat 3.5 software (SigmaStat software, San Jose, CA, USA). After testing data for normal distribution, the appropriate statistical test was used and the overall *p* value indicated in the figure legends. Group differences were considered significant when *p* was < 0.05, indicated by an asterisk; those at *p* was < 0.01 are indicated by double asterisks; those at *p* < 0.001 are indicated by triple asterisks. For the Raman spectra, Principal Component Analysis and Linear Discriminant Analysis (PCA-LDA) were performed by means of Origin2018 (OriginLab, Northampton, MA, USA). To test the sensitivity, specificity and accuracy of the classification model to distinguish the EV phenotype by the overall biochemical composition, leave-one-out cross-validation was used.

## Results

### EVs bud at the surface of microglia infiltrating the myelin lesion

Injection of the detergent lysolecithin into the mouse corpus callosum (CC) causes a demyelinating injury within 4 days which spontaneously recovers over time, offering a robust approach to study remyelination [64, 69]. In this model, infiltration and activation of macrophage/microglia were shown to be important for OPC migration and successful differentiation into myelin-forming cells [50, 64].

**Fig. 1:**
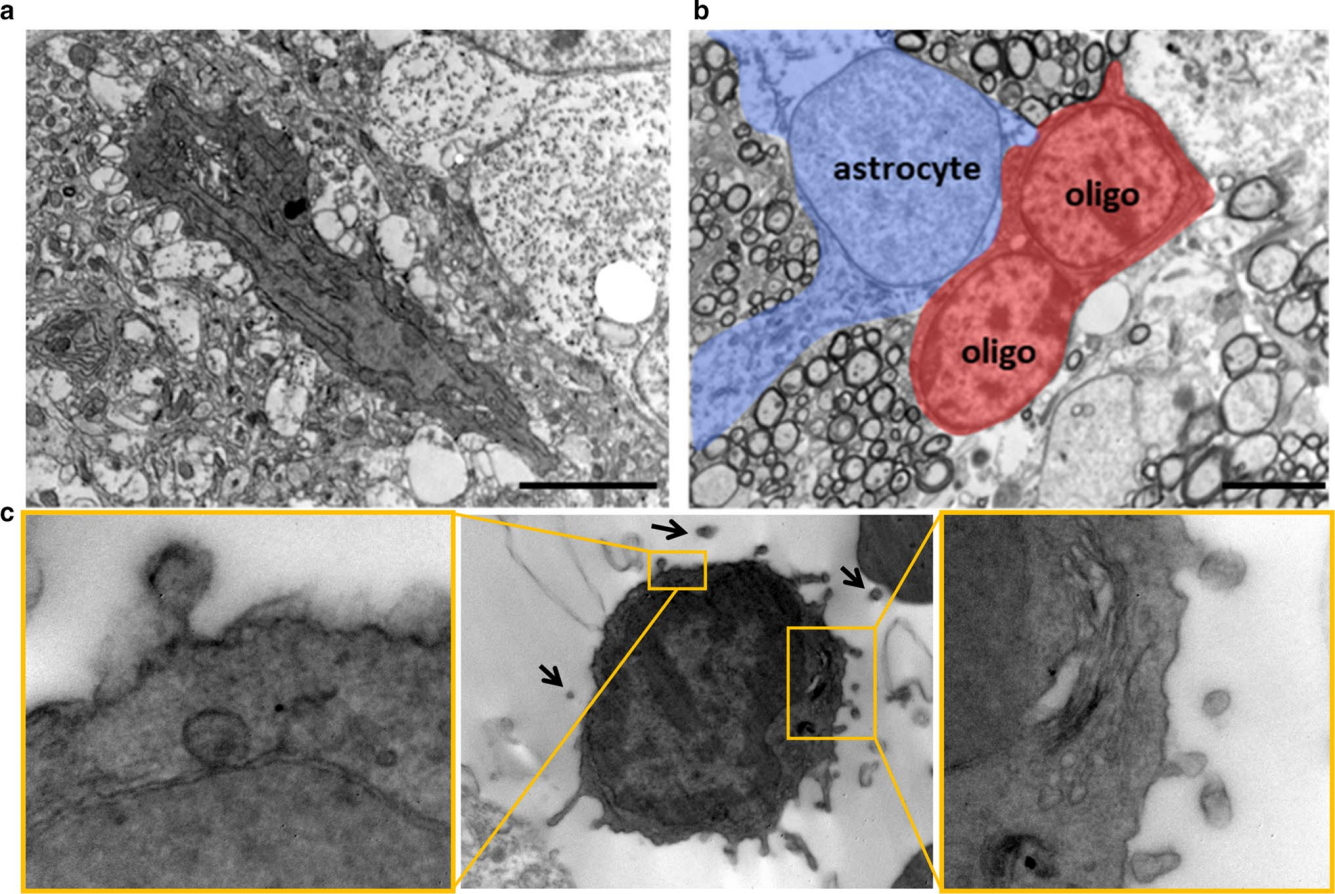
Microglia infiltrating the myelin lesion release EVs. EM images of the CC showing dark cells resembling microglia (**a**), oligodendrocytes (**b**) and astrocytes (**b**) infiltrating demyelinated lesion at 10 dpl. High magnification inserts in **c** show examples of EVs budding from the surface of dark microglia (scale bars 2 μm)

Ten days after the lesion (dpl), through high-resolution EM analysis, we found cells with the ultrastructural features of microglia (Fig. 1a) along with astrocytes and oligodendrocytes (Fig. 1b) at the lesion site. Importantly, microglia exhibited condensed, electron-dense cytoplasm and nucleoplasm, resembling “dark” microglia, a recently described phenotype of reactive cells [7, 27], characterized by pronounced chromatin remodelling, which is indicative of altered gene expression. Although the narrow conformation of the extracellular space commonly constrains EV visualisation, we observed a dark microglial cell infiltrating the lesion, with several blebs at the cell surface and surrounded by putative membrane vesicles in the pericellular space (Fig. 1c), suggesting intense EV production. This evidence prompted us to investigate the role of EVs in the interplay between reactive microglia and OPCs at lesion sites. Inhibiting endogenous EV production without interfering with the remyelination process is challenging, given that most effective pharmacological or genetic inhibitors of EV biogenesis interfere with sphingolipid metabolism [94]. We, therefore, analysed the action of exogenous EVs produced in vitro by polarised microglia.

### Distinct phenotypic signatures of EVs released by either pro-inflammatory or pro-regenerative microglia

**Fig. 2:**
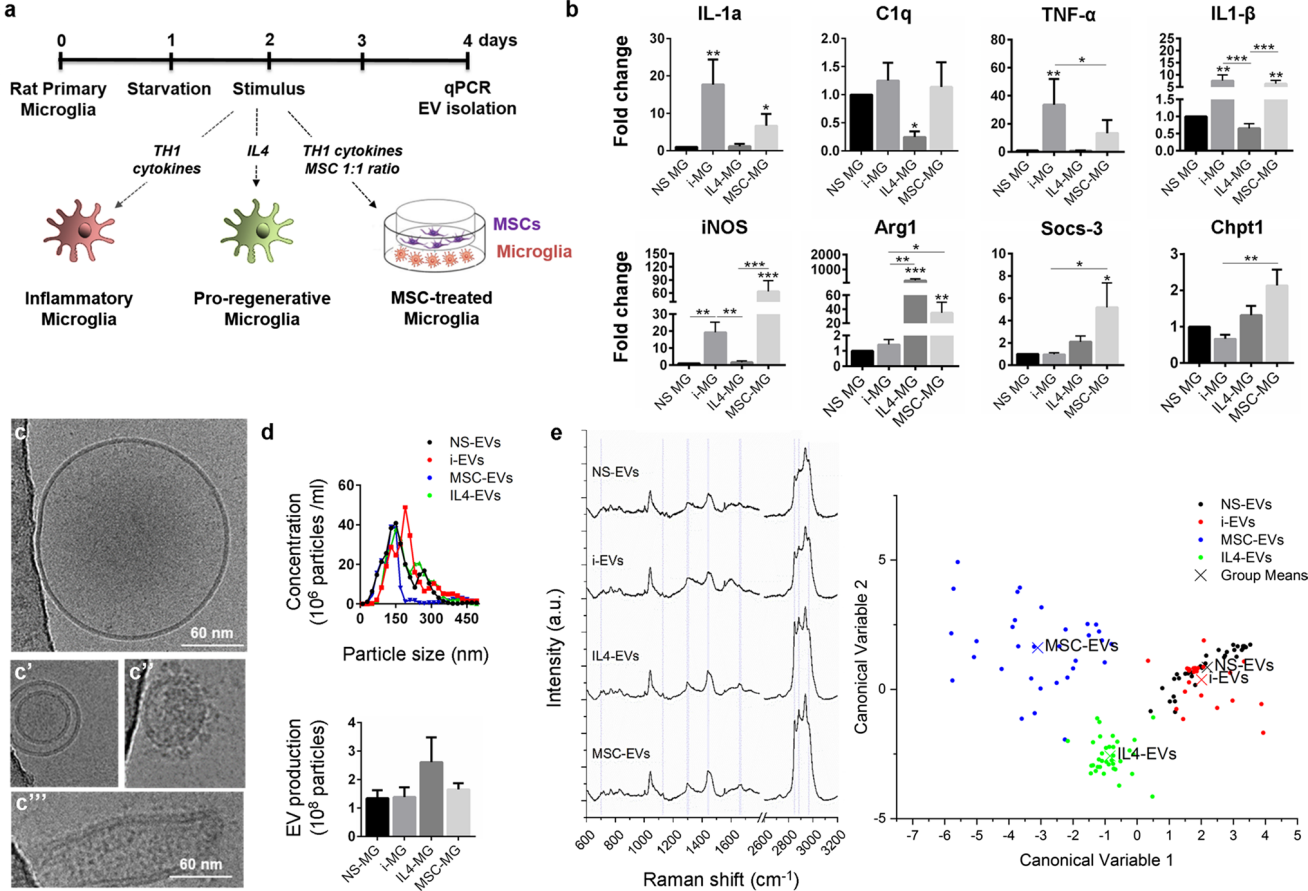
Production and characterization of EVs released by microglia with different activation states. **a** Scheme of microglia polarisation in vitro. **b** Gene expression of inflammatory markers (IL-1a, C1q, TNF-α, IL-1β and iNOS), pro-regenerative markers (Arg1 and Socs-3) and the metabolic gene Chpt1 in unstimulated microglia (NS-MG), IL-4-treated microglia (IL-4-MG) or microglia stimulated with inflammatory cytokines in the absence (i-MG) or in the presence of MSCs (MSC-MG) [number of independent experiments (*n*) = 7–11/group; Kruskal–Wallis test with Dunn’s multiple comparison *p < 0.05, **p < 0.01, ***p < 0.001. **c** Representative cryo-EM images of the heterogeneous population of microglial EVs in the 100 k g pellet. **d** Size profile of EVs pelleted from 1 × 10^6^ microglia, re-suspended in 100 μl of 0.1 µm-filtered PBS and analysed using NTA (top). Histograms show production of EVs from NS or polarised microglia during 30 min stimulation with 1 mM ATP (bottom) (*n* = 4; one way ANOVA *p* = 0.2898 with Tukey’s multiple comparisons test]. **e** Mean Raman spectra obtained using 532 nm laser line from EVs of NS or polarized microglia. All spectra were baseline corrected, aligned and normalised before averaging. **f** Multivariate statistical analysis performed on the Raman spectra (*n* ≥ 30 per sample). The scatter plot represents the values obtained for the Canonical Variable 1 and Canonical Variable 2 after LDA. In the classification model, spectra from EVs were grouped based on the cell of origin to test RS ability to discriminate the molecular composition of EVs from different microglial phenotypes. The first 10 PC scores calculated by means of PCA were used for the LDA. Each dot represents a single spectrum

Primary rat microglia were maintained in the absence of stimuli (NS), under inflammatory (Th1 cytokine cocktail) or pro-regenerative (IL-4) conditions for 48 h. They were also co-cultured with MSCs in transwell system in the presence of inflammatory cytokines (Fig. 2a) to drive pro-regenerative functions [36, 101]. Gene expression analysis revealed distinct patterns of phenotypic marker expression in the four conditions. Despite common upregulation of pro-regenerative arginase 1 (Arg1) in MSC-treated and IL-4-treated microglia, the former ones maintained pro-inflammatory traits (elevated IL-1a, C1q, IL-1β and iNOS) and selectively upregulated a few genes, including Suppressor of cytokine signaling 3 (Socs-3), a gene involved in negative regulation of cytokines and Choline Phosphotransferase 1 (Chpt1), an enzyme involved in phosphatidylcholine synthesis (Fig. 2b).

Non-stimulated (NS) microglia and polarised cells were exposed to ATP for 30 min to promote EV release, and EVs were isolated by ultracentrifugation at 100 kg after pre-clearing of cell supernatants from cells and debris [34]. Cryo-electron microscopy (cryo-EM) analysis revealed that most EVs from NS microglia consisted of a single lipid bilayer (Fig. 2c, c″, c‴; 88%) and were rounded in shape (Fig. 2c–c″; 66%), but were very heterogeneous in size, ranging from 15 to 1070 nm in diameter (mean diameter 81.14 ± 9.11 nm). A subpopulation of EVs contained one or more vesicles in their lumen (10%; Fig. 2c′), or were coated on their surface (Fig. 2c″; 15%), likely due to the presence of transmembrane or membrane-associated proteins. According to our previous evidence [34, 95], cryo-EM excluded contamination with apoptotic bodies or intracellular organelles derived from damaged cells. Nanosight Tracking Analysis showed no major changes in total EV production from reactive microglia compared to NS cells (Fig. 2d). Conversely, clear differences in the molecular composition of EVs derived from polarised microglia were revealed by Raman spectroscopy (Fig. 2e), a sensitive optical technique already proven to provide information on the chemical content of EVs [39, 40, 51, 85, 88]. Beside the characteristic bands of proteins and nucleic acids (proteins: Amide I 1600–1690 cm^−1^, Amide III 1200–1300 cm^−1^; nucleic acids: 720–820 cm^−1^), main differences were identified in the spectral intervals that can be attributed to lipid components (mainly 2700–3200 cm^−1^). In particular, EVs derived from non-stimulated and polarised microglia showed differences in the relative intensities of peaks attributable to cholesterol and cholesterol ester (700; 1127; 1440 cm^−1^) and in peaks corresponding to the CH_2_ deformations (around 1300 cm^−1^) and CH, CH_2_, and CH_3_ bonds (in the spectral range 2600–3200 cm^−1^), which are known to be related to lipids [43]. Despite such similarities, significant differences among IL4-EVs and MSC-EVs spectra were highlighted by linear discrimination analysis, suggesting that the two types of EVs carry distinct cargoes beyond common molecules, thus reflecting the distinct phenotypes of donor cells. The classification model PCA-LDA demonstrated that RS can distinguish the microglia phenotypes of the EV source with an overall accuracy of 91.7% and an error rate of 18.24% after cross-validation. The error rate was reduced to 10.16% when analysing spectra from broken EVs, busted by hypo-osmotic shock [34] and re-pelleted to remove soluble components supporting the major contribution of membrane components in the spectral differences between the EV subtypes (Suppl. Figure 1, Online Resource).

### Microglia-derived EVs display a dual action on the oligodendroglial response to focal demyelinating lesions

**Fig. 3:**
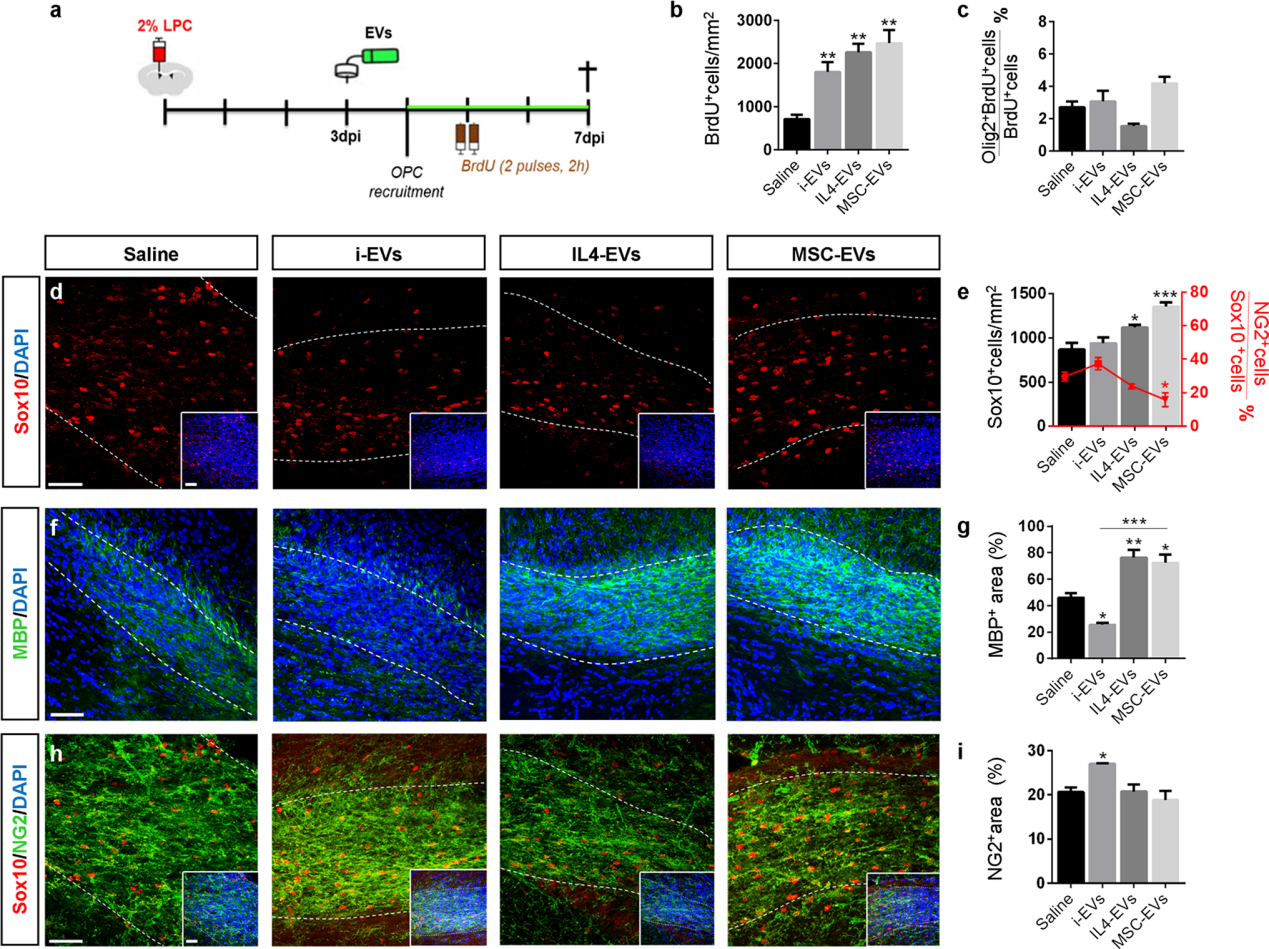
Action of EVs on early response to EVs. **a** Experimental design of EV delivery to LPC-treated mice during the phase of OPC recruitment. Histograms show the density of total proliferating cells (**b**) [number of animals (*N*) = 3–5/group; one-way ANOVA main effect of treatment *p* = 0.0021 with Holm–Sidak’s multiple comparisons test vs Saline] and the percentage of BrdU^+^ proliferating OPCs (**c**) (*N* = 3–5/group; one way ANOVA *p* = 0.0165 with Holm–Sidak’s multiple comparisons test vs Saline] in saline, i-EVs- IL4-EVs- or MSC-EVs-injected lesions. **d** Representative images of Saline, i-EVs, IL4-EVs or MSC-EVs-injected lesions (area delimited by dotted line) immunostained against Sox10 (red) (scale bars of images and insets, 50 μm). Low magnification inserts show Sox10/DAPI double staining to visualise nuclei. **e** Corresponding density of Sox10^+^ cells (histograms) (*N* = 3–5/group; one way ANOVA *p* = 0.0013 with Holm-Sidak’s multiple comparisons test vs Saline) and percentage of immature (NG2^+^ Sox10^+^) oligodendrocytes (red line) (*N* = 3–5/group; one way ANOVA *p* = 0.0074 with Holm–Sidak’s multiple comparisons test vs Saline). **f–h** Representative images of saline, i-EVs, IL4-EVs or MSC-EVs-injected lesions (area delimited by dotted line) immunostained against MBP (green) and DAPI (blue) (**f**) or against NG2 (green), Sox10 (red) and DAPI (blue) (**h**) (scale bars 50 μm). **g**, **i** Histograms show the percentage of the lesioned area immunoreactive for MBP (**g**) (*N* = 3–7/group; one way ANOVA *p* = 0.0001 with Holm–Sidak’s multiple comparisons test vs Saline) or NG2 (**i**) (*N* = 3–4/group; one way ANOVA *p* = 0.0173 with Holm–Sidak’s multiple comparisons test vs Saline) in saline-injected mice and mice that received different types of EVs

We first examined the action of EVs produced by inflammatory microglia (i-EVs) and pro-regenerative cells (IL4-EVs and MSC-EVs) on the early response to demyelination (3 dpl), during the phase of OPC recruitment [41, 48, 64]. EVs produced by 1.5 million cells (~ 2–3 × 10^8^) were delivered to focal myelin lesion via pre–filled Alzet mini-pumps for 4 days (at 2 × 10^6^ EVs h^−1^). EVs were still present in the mini-pumps 3 days after re-suspension, albeit the concentration was reduced by ~ 30%, as measured by Nanosight Tracking Analysis (EV concentration = 2.80 × 10^8^ ± 0.19 at day 0; 2.60 × 10^8^ ± 0.13 at day 1; 1.90 × 10^8^ ± 0.08 at day 3). Mice received BrdU twice (2 h apart), at 4 dpl, to monitor cell proliferation and the tissue was analysed at the end of the treatment (7 dpl) (Fig. 3a).

Quantification of BrdU^+^ cells showed that all EV types significantly increased cell proliferation in the lesion area compared to saline-injected controls (Fig. 3b). However, we found almost no impact of EVs on the proliferation of Olig2^+^ oligodendroglial cells (Fig. 3c), indicating that other brain cells proliferate in response to EVs. While not increasing oligodendrocyte proliferation, both IL4-EVs and MSC-EVs increased the density of oligodendroglial cells positive for the transcription factors Sox10 in the lesion area, suggesting enhanced migration of OPCs to the lesion site (Fig. 3d, e). Staining for myelin protein MBP showed that both IL4-EVs and MSC-EVs promoted the full differentiation of oligodendrocytes, as shown by increased MBP^+^ labelling in the lesioned area (Fig. 3f, g). This maturation effect was accompanied by a decrease in immature NG2 + OPCs (NG2^+^/Sox10^+^ cells) (Fig. 3e) and an increase in density of oligodendrocytes undergoing differentiation (NG2^−^Sox10^+^ cell density: saline, 721.3 ± 88.64; i-EVs, 562.0 ± 40.31; IL4-EVs, 755.1 ± 82.13; MSC-EVs, 1080 ± 98.97; one-way ANOVA main effect of treatment *p* = 0.0114 with Holm-Sidak’s multiple comparisons test) upon MSC-EVs delivery. By contrast, the fraction of MBP staining in the lesioned area decreased upon i-EV infusion (Fig. 3f, g) while the tissue exhibited a significant increase in NG2 staining (Fig. 3h, i). Accordingly, the percentage of NG2^+^/Sox10^+^ early progenitors increased in comparison to controls (Fig. 3e).

Overall, these data indicate that prolonged delivery of EVs produced by beneficial microglia, especially MSC-treated microglia, promotes the accumulation of oligodendrocytes at the lesion site and enhances remyelination, whereas i-EVs injection inhibits OPC maturation.

**Fig. 4:**
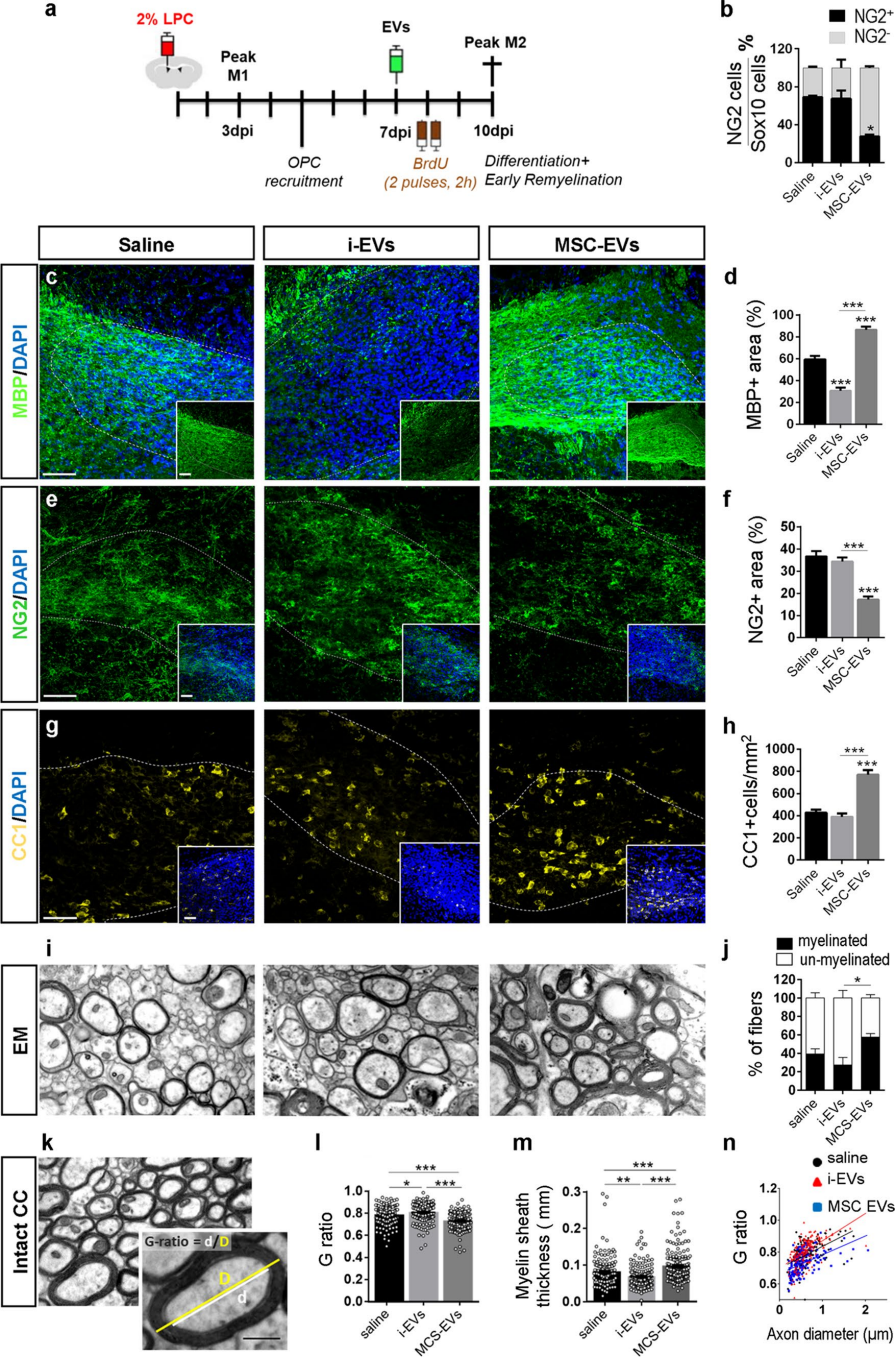
Action of EVs on myelin deposition in acute LPC-mediated focal demyelination. **a** Experimental protocol of EV delivery to LPC-treated mice during the phase of OPC differentiation. **b** Histograms show percentage of immature (Sox10^+^NG2^+^) and differentiated cells (Sox10^+^NG2^−^), oligodendrocytes in saline-injected mice and mice that received i-EVs or MSC-EVs (*N* = 3–4/group; Kruskal–Wallis test *p* = 0.0129 with Dunn’s multiple comparisons test vs Saline). Representative images of saline, i-EVs or MSC-EVs-injected lesions (area delimited by dotted line), immunostained against MBP (green, **c**), NG2 (green, **e**), or CC1 (yellow, **g**) (scale bars 50 μm). Histograms show the percentage of the lesioned area immunoreactive for MBP (**d**) (*N* = 3–4/group; one-way ANOVA *p* < 0.0001 with Holm–Sidak’s multiple comparisons test), NG2 (**f**) (*N* = 4/group; one-way ANOVA *p *< 0.0001 with Holm–Sidak’s multiple comparisons test) or the density of mature oligodendrocytes (CC1) (**h**) (*N* = 3/group; one-way ANOVA *p* < 0.0001 with Holm–Sidak’s multiple comparisons test. **i** Representative electron micrographs of CC in saline-injected mice and mice that received i-EVs or MSC-EVs (scale bars of images, 1 μm; original magnification 25,000). **j** Histograms show the percentage of unmyelinated/myelinated fibres (*N* = 3/group; one-way ANOVA *p* = 0.039 with Dunn’s multiple comparison test). **k** Myelin thickness can be quantified by the *G*-ratio, defined as the ratio between the inner (axonal, **d**, white) and outer (overlying myelin, **d**, yellow) diameters of myelinated axons. Scale bar of image, 1.5 μm. **l** Histograms show quantifications of the *G*-ratio (*N* = 3/group; Kruskal–Wallis test *p* < 0.0001 with Dunn’s multiple comparisons test). **m** Histograms show myelin sheath thickness (*N* = 3/group; Kruskal–Wallis test *p* < 0.0001 with Dunn’s multiple comparisons test). Each dot represents an individual value. **n** Scatter plots of *G*-ratio against axon diameter. The *G*-ratio of each measured myelinated fibre is indicated by a single circle (*n* = 160 saline, *n* = 167 MSC-EVs, *n* = 169 i-EVs). Correlation between axon diameter (x1) and *G*-ratio (y1) is expressed by the correlation coefficient (*r*) of the linear regression curve (saline: *r* = 0.503, i-EVs: *r* = 0.518; MSC-EVs: *r* = 0.448)

To further assess the impact of EVs on OPC differentiation, MSC-EVs and i-EVs (7 × 10^7^ EVs, corresponding to about one third of EVs delivered over 4 days via the minipump) were delivered to LPC-treated mice by a single injection at 7 dpl, when OPCs become post-mitotic and progress toward differentiation into myelin-sheath forming oligodendrocytes [64]. The remyelination process was analysed at 10 dpl in both young (2.5–4 months old) and aged (8–12 months old) mice (Fig. 4a), in which remyelination occurs more slowly [44, 82–84]. MRI analysis showed that this time-window is optimal to study remyelination in our experimental setting. Indeed, at 5–6 dpl, before EV treatment, hyper-intense signal on T2-weighted MR images indicated clear myelin lesions in almost the entire CC, whereas at 10 dpl CC signal returned hypointense almost similar to healthy controls, suggesting fast recovery (Suppl. Figure 2, Online Resource). Remyelination of CC was also observed by diffusion tensor imaging (DTI), showing considerably increased diffusivity perpendicular to white matter fibres at 5 dpl, and return to normal values at 10 dpl (Suppl. Figure 2, Online Resource).

The presence of EVs at the lesion site was verified in mice injected with GFP-labelled EVs produced by GFP-expressing microglia. Twenty minutes after a single injection, GFP-EVs were localised within the lesion evidenced by DAPI staining (Suppl. Figure 3a–d′, Online Resource). Immunolabelling for the lineage markers Iba1, S100β or NG2 and 3D reconstruction by Imaris showed GFP-EVs inside microglia/macrophages (Suppl Fig. 3e–e′‴, Online Resource), astrocytes (Suppl Fig. 3f–f″, Online Resource) or NG2^+^ cells (Suppl Fig. 3 g–g‴, Online Resource). 60 min after injection, GFP-EVs were no longer visible in proximity or away from the injury (not shown). Despite being injected in a single-pulse and in lower amounts compared to the prolonged treatment, both i-EVs and MSC-EVs had a strong impact on oligodendrocytes during the phase of OPC differentiation. In aged mice, i-EVs produced a significant decrease in MBP staining (Fig. 4c, d) with no changes in the fraction of NG2 staining in the lesioned area (Fig. 4e, f) and in the percentage of NG2^+^ early progenitors (Fig. 4b). Compared to saline and i-EV injected animals, mice receiving MSC-EVs showed enhanced MBP staining (Fig. 4c, d) and decreased NG2 labelling (Fig. 4e, f), consistent with OPC differentiation into myelin-sheath forming oligodendrocytes. These results were corroborated by the increase in the density of oligodendrocytes positive for CC1, a myelinating oligodendrocyte marker that stains the cell body [6] (Fig. 4g, h), and by the decrease in the percentage of NG2^+^/Sox10^+^ early progenitors at lesion site (Fig. 4b).

**Fig. 5:**
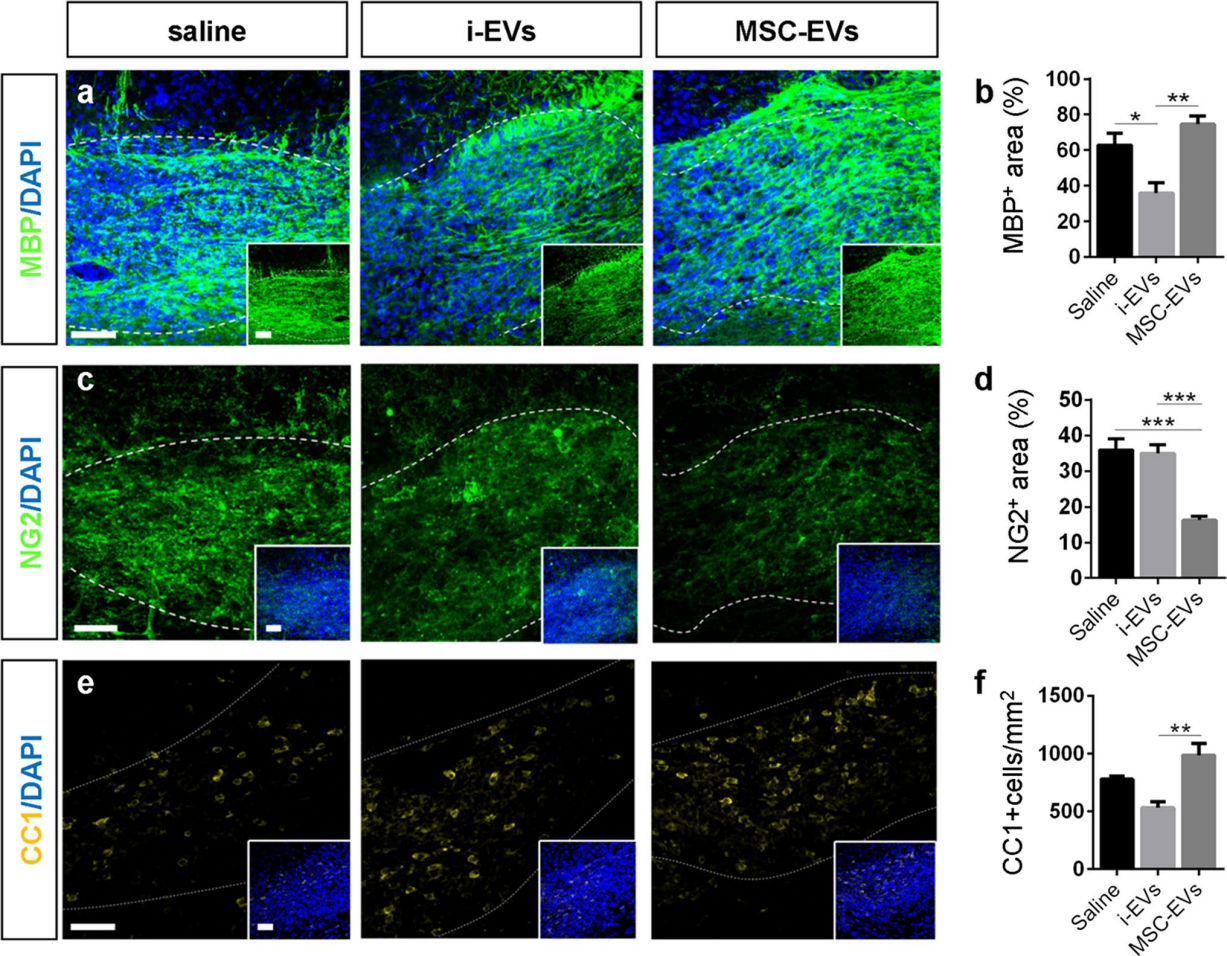
Action of microglia-derived EVs on OPC differentiation at myelin lesion in young mice. Representative images of saline, i-EVs and MSC-EVs-injected lesions (area delimited by dotted line), immunostained against MBP (green, **a**), NG2 (green, **c**), or CC1 (yellow, **e**) (scale bars 50 μm). Low magnification inserts show double labelling for DAPI. Histograms show the percentage of the lesioned area immunoreactive for MBP (**b**), NG2 (**d**) or CC1 (**f**) at 7 dpl in saline-injected mice and mice injected with i-EVs or MSC-EVs [number of animals (*N*) = 3–5/group, one-way ANOVA with Holm–Sidak’s multiple comparison: MBP, *p* = 0.0012; NG2, *p* < 0.0001; CC1, *p* = 0.0090)

In young mice, i-EVs also exerted an inhibitory action on OPC maturation, as indicated by decreased MBP staining at the lesion site (Fig. 5a, b), while the effect of MSC-EVs was less evident. Compared to i-EVs, MSC-EVs significantly increased MBP immunoreactivity (Fig. 5a, b), decreased NG2 staining (Fig. 5c, d) and enhanced the density of CC1^+^ differentiated oligodendrocytes (Fig. 5e, f), but failed to increase remyelination over control levels (Fig. 5a, b, e, f).

Altogether, these data indicate that, while inflammatory microglia inhibit OPC differentiation and remyelination through secretion of i-EVs, MSC-treated microglia release EVs which favor recruitment and transition of NG2 cells into mature myelinating oligodendrocytes at the lesion site.

### EVs from different sources induce qualitatively and quantitatively different myelin ultrastuctural features

For a more detailed definition of myelin ultrastructure, we evaluated the remyelination process by EM analysis at 10 dpl in the CC of aged lesioned mice that received either i-EVs, or MSC-EVs or saline by a single injection. i-EVs-injected mice displayed a higher G ratio (Fig. 4k–m) and thinner myelin (Fig. 4l) compared to saline-injected mice, confirming that i-EVs hamper remyelination. By contrast, mice injected with MSC-EVs exhibited a higher percentage of myelinated fibres (Fig. 4i, j), lower G ratio (Fig. 4k–m) and higher myelin sheath thickness (Fig. 4l) compared to saline control samples and i-EVs-injected mice, consistent with a promoting action of MSC-EVs on myelin repair.

### Strong impact of EVs on OPC migration: the role of sphingosine-1-phosphate

To investigate the molecular mechanisms underlying harmful and protective action of microglia-derived EVs, we set up primary OPC cultures, a powerful system with easy access for EVs, which allows to dissect their direct effects. All in vitro experiments were performed by exposing OPCs to EVs produced by twice as many microglial cells, in order to keep conditions close to physiology. To further explore how the differential activation state of microglia influences EV action, NS-EVs were included in the analyses.

**Fig. 6:**
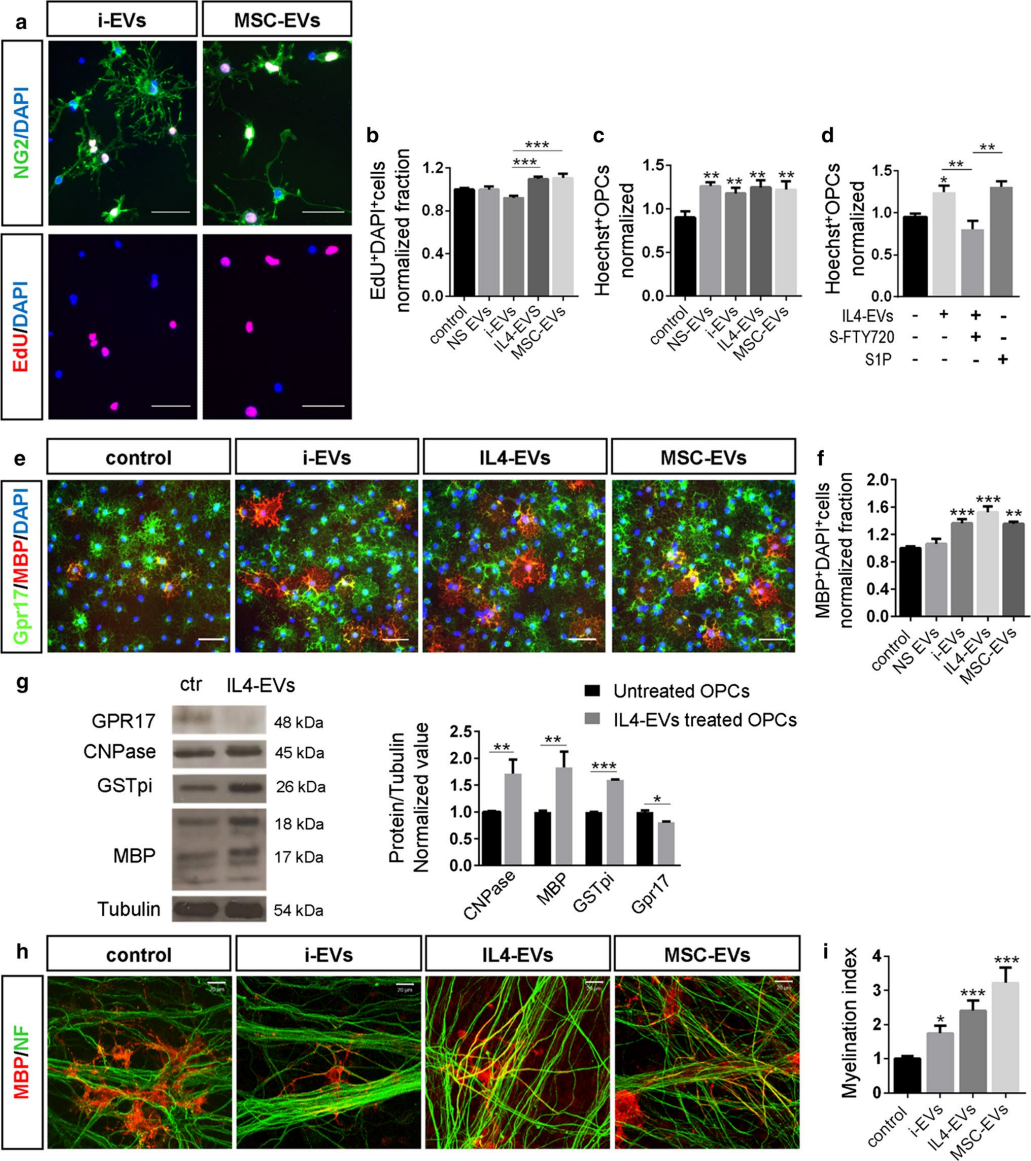
EV impact on OPC migration, differentiation and myelination. **a**, **b** Fluorescence images of cultured OPCs incubated with EdU (red), fixed and stained for NG2 (green) and DAPI (blue) after 24 h exposure to i-EVs or MSC-EVs (scale bars 50 µm). The histograms in **b** show the percentage of EdU^+^ OPCs in cultures exposed or not to different EV types. Data have been normalized to control [number of experiments (*n*) = 5–8/group; Kruskal–Wallis test *p* < 0.0001 with Dunn’s multiple comparisons test]. **c** Histograms show the percentage of OPCs migrated through the filter of the Boyden chamber in control conditions and following addition of different types of EVs. Data have been normalized to control (*n* = 3; one-way ANOVA *p* = 0.0054 with Holm–Sidak’s multiple comparisons test vs control). **d** Percentage of migrated OPCs in response to S1P or IL-4-EVs in the presence or in the absence of the S1P receptor antagonist S-FTY720-Vinylphosphonate (*n* = 3; Kruskal–Wallis test *p* < 0.0001 with Dunn’s multiple comparisons test). **e** Representative images of OPCs maintained in control conditions or exposed to different types of EVs for 2 days, fixed and stained for MBP (red), GPR17 (green) and DAPI (blue) (scale bars 50 µm). **f** Corresponding quantification of MBP^+^ OPCs. Data have been normalized to control (*n* = 5–8/group, Kruskal–Wallis test *p* < 0.0001 with Dunn’s multiple comparisons test vs control). **g** Western blot of control OPCs and IL4-EVs-treated OPCs for the indicated markers of OPC differentiation. Tubulin has been used as loading control. Relative quantification of the band density is shown on the right (*n* = 3; CNPase: unpaired *t* test *p* = 0.0033; MBP: unpaired *t* test *p* = 0.0018; GST-pi: unpaired *t* test *p* < 0.0001; GPR17: unpaired *t* test *p* = 0.0224). **h** Representative images of OPC-DRG co-cultures maintained in control conditions or exposed to i-EVs, IL4-EVs or MSC-EVs for 11 days, fixed and stained for MBP (red) and neurofilament (NF, green) (scale bars 20 µm). **e** Myelination index (MBP staining/NF staining) under different experimental conditions (*n* = 3; Kruskal–Wallis test *p* < 0.0001 with Dunn’s multiple comparisons test vs control)

First, we explored whether EVs have the capacity to influence OPC proliferation and/or migration, two mechanisms involved in oligodendrocyte recruitment into focal myelin lesion. The impact of EVs on proliferation was assessed by exposing cultured OPCs to the different types of EVs for 24 h in the presence of the proliferative marker EdU. Quantitative analysis of EdU^+^ OPCs showed that i-EVs reduce OPC proliferation, albeit with no statistical significance, while EVs produced by pro-regenerative microglia (either IL4-EVs or MSC-EVs) significantly increased OPC proliferation compared to i-EVs-treated, but not to NS-EVs or untreated (control) OPCs (Fig. 6a, b). In agreement with in vivo observations, these data show very mild effects of EVs on OPC proliferation. By contrast, the impact of EVs on OPC migration was much stronger. Using a classical transwell-based migration assay, we found that all types of EVs significantly enhance the transit of OPCs through the transwell filter, regardless of the activation state of parent microglia (Fig. 6c).

EVs contain components of the sphingolipid pathway, which play a key role in biogenesis and biological action of EVs [94]. Among them, sphingosine-1-phosphate (S1P) is a known chemoattractant agent [49], which may be involved in OPC migration. To test this hypothesis, we pulse-labelled microglia with [^3^H]sphingosine ([^3^H]sph) under conditions of metabolic equilibrium and measured the content of [^3^H]sphingolipids in EVs and parental cells. We found detectable levels of [^3^H]S1P along with more abundant sphingolipids (sphingomyelin, lactosylceramide and monosialodihexosylganglioside (GM3) in EVs produced by NS microglia, but S1P was not enriched in EVs compared to donor cells (Table 3). We next monitored OPC migration in the presence of the pan S1P receptor antagonist S-FTY720-Vinylphosphonate (100 nM) [92] and found that the drug completely inhibited the capacity of EVs to attract OPCs (Fig. 6d).

**Table 3:** Sphingolipid content in EVs and parental microglia

Sphingolipid species	% in cells	% in EVs	EVs/cell fold increase
[^3^H]Sphingomyelin	27.55 ± 2.4	33.88 ± 3.4	1.2
[^3^H]Ceramide	4.04 ± 0.4	4.60 ± 0.4	1.1
[^3^H]Glucosylceramide	5.97 ± 0.5	4.41 ± 0.4	0.7
[^3^H]Lactosylceramide	19.26 ± 1.8	11.41 ± 1.4	0.6
[^3^H]Sphingosine	3.78 ± 0.4	4.31 ± 0.5	1.1
[^3^H]GM3	10.4 ± 0.6	8.94 ± 1.0	0.8
[^3^H]Sphingosine-1-phosphate	1.17 ± 0.14	0.95 ± 0.15	0.8

Collectively, these results indicate that microglial EVs promote OPC migration and highlight a role for vesicular S1P as attractive guidance cue for OPCs.

### In vitro, EVs derived from both inflammatory and pro-regenerative microglia promote OPC differentiation

To define direct effects of EVs on OPC differentiation, cells were incubated with the different types of EVs for 48 h, fixed and stained for MBP. Unexpectedly, immunofluorescence analysis revealed that i-EVs significantly enhanced the fraction of MBP^+^ oligodendrocytes in a similar way to IL4-EVs and MSC-EVs, whereas NS-EVs did not influence OPC maturation (Fig. 6e, f). No significant changes in the fraction of MBP^+^ cells were observed with half or double concentration of IL4-EVs, excluding the possibility that small variations in EV amount could have had a strong impact on OPC maturation (normalized MBP^+^ OPC fraction: 1.53 ± 0.26 standard EV dose; 1.42 ± 0.22 half dose; 1.27 ± 0.36 double dose). Western blot analysis confirmed that IL4-EVs enhance the expression of MBP along with other markers of mature oligodendrocytes, i.e. 2′,3′-cyclic nucleotide-3′-phosphodiesterase (CNPase) and glutathione S-transferase (GST)-pi while downregulating GPR17, a marker of immature oligodendrocytes (Fig. 6g).

Direct action of EVs on myelin deposition was investigated in OPCs co-cultured with DRG neurons, a useful system to study myelination [16]. OPCs were exposed to EVs for 11 days (three treatments, every 3/4 days), fixed and immunostained with axon- and myelin-specific markers, i.e. high-molecular-weight neurofilaments (NF) and MBP, respectively. Quantitative analysis of the linear MBP^+^ segments extending along axons showed that both IL-4-EVs and MSC-EVs favoured myelin deposition (Fig. 6h, i). However, i-EVs also significantly enhanced myelination, albeit at a lower extent compared to pro-regenerative EVs. NS-EVs had no significant effect on myelination (not shown). Of note, MSC-EVs displayed higher capacity to promote myelin deposition compared to EVs released by MSCs (Suppl. Figure 4a, Online Resource).

Globally, these results show that i-EVs exhibit direct pro-differentiating action on cultured OPCs. Thus, the blockade of OPC maturation caused by i-EVs in vivo likely involves other brain cells, which may acquire harmful function in response to i-EVs.

### Astrocytes mediate the harmful effects of i-EVs on OPCs

**Fig. 7:**
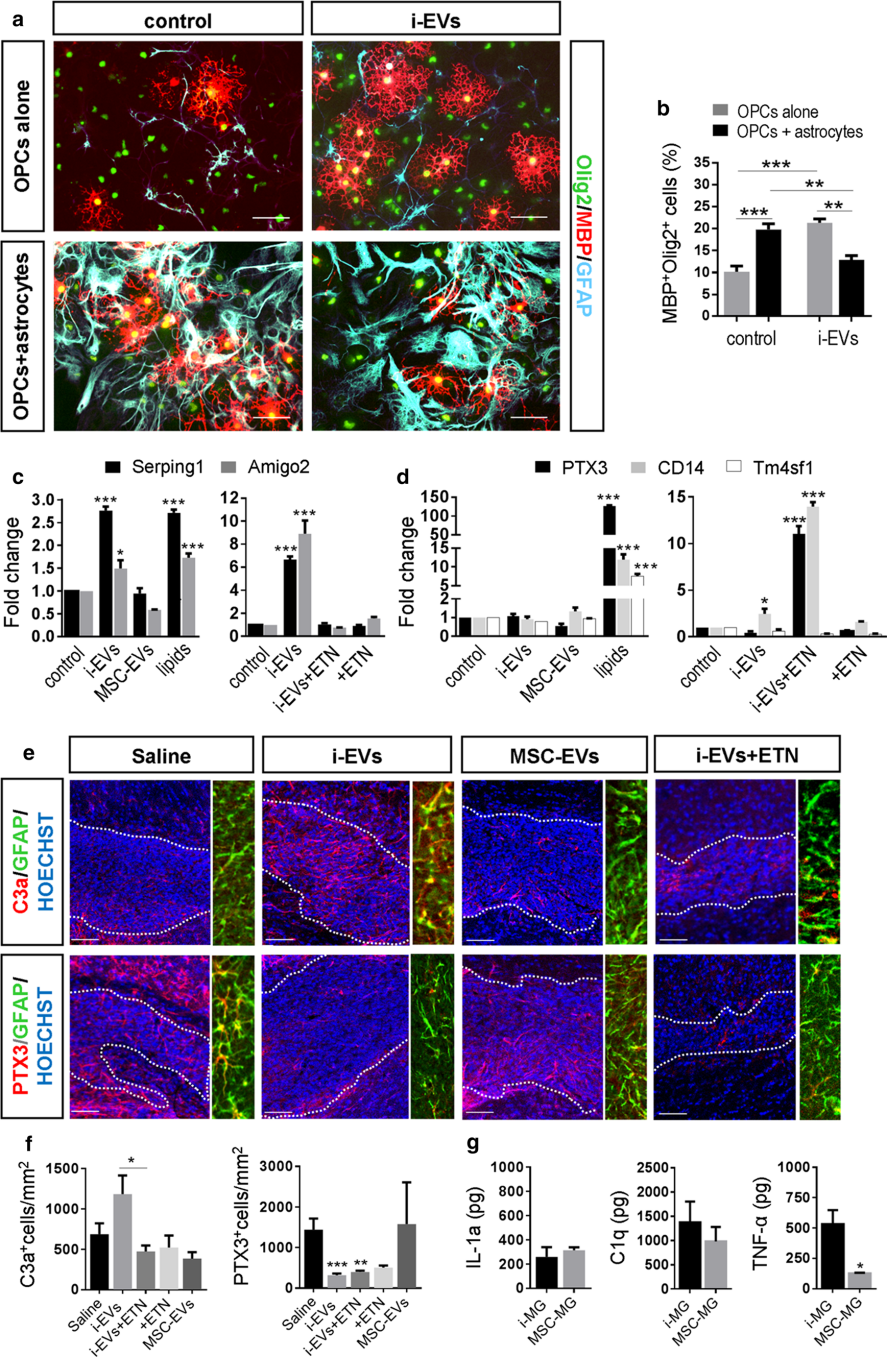
Astrocytes transform the pro-differentiating action of i-EVs to inhibitory activity. **a** Representative images of OPCs cultured alone or with astrocytes in the presence or in the absence of i-EVs immunostained for MBP (red), Olig2 (green) and GFAP (cyan) (scale bars 20 μm). **b** Corresponding quantifications of MBP^+^ OPCs (*n* = 2; one-way ANOVA *p* < 0.0001 with Tukey’s multiple comparisons test). Representative qPCR analysis of A1 (**c**) and A2 (**d**) marker expression in unstimulated astrocytes (control), astrocytes exposed to i-EVs, the lipid fraction of i-EVs (lipids), MSC-EVs (left panels). Right panels show A1 and A2 marker expression in astrocytes exposed to i-EVs in the presence/absence of the TNF-a inhibitor etanercept (ETN).Three replicates/condition have been normalized to control (one-way ANOVA *p* < 0.0001 with Holm–Sidak’s multiple comparisons test). **e** Representative images of saline, i-EVs, or MSC-EVs-injected lesions, immunostained against C3 or PTX3 (red) and Hoechst (blue) (scale bars 50 μm). High magnification inserts show astrocytes double stained for GFAP (green) and C3 or PTX3 (red). **f** Density of C3- and PTX3-positive astrocytes at saline, i-EVs-, i-EVs + ETN, or MSC-EVs-injected lesions (C3, number of sections = 5–10/group; Kruskal–Wallis test *p* = 0.0298 with Dunn’s multiple comparisons test among Saline, i-EVs and i-EVs + ETN); (PTX3, number of sections = 5–10/group; Kruskal–Wallis test *p* < 0.0001 with Dunn’s multiple comparisons test). **g** ELISA quantification of IL-1a, C1q, and TNF-α in 1X10^6^ inflammatory microglia (i-MG) and in MSC-treated microglia (MSC-MG) (*n* = 3; IL-1a, Mann–Whitney *t* test *p* = 0.9000; C1q, unpaired t test *p* = 0.5619; TNF-α, Mann–Whitney *t* test *p* = 0.0286)

Recent evidence indicates that microglia can transform astrocytes into A1 harmful cells, which inhibit OPC differentiation [99]. To investigate the possible involvement of astrocytes in the detrimental action of i-EVs, we grew OPCs on top of astrocytes and maintained the co-culture in the presence or absence of i-EVs for 48 h. Immunofluorescence staining for MBP revealed that, in the absence of microglial i-EVs, astrocytes accelerated OPC differentiation, as indicated by higher percentages of MBP/Olig-2 double-positive cells in astrocyte-OPC co-cultures compared to OPCs cultured alone (Fig. 7a, b). However, when exposed to i-EVs, astrocytes caused a strong inhibition of OPC maturation (Fig. 7a, b). Consistent with harmful astrocyte transformation, qPCR analysis showed that, in response to i-EVs, pure cultured astrocytes upregulated the A1 reactive markers serping-1 and Amigo 2 [55] (Fig. 7c), whilst exhibiting unaltered expression for the A2 markers pentraxin-3 (PTX3), CD14 and Tm4sf1 (Fig. 7d). In contrast, the lipid extract of i-EVs potently induced A2 markers (Fig. 7d), and slightly increased serping-1 and Amigo 2 (Fig. 7c), suggesting a shift of astrocytes towards beneficial rather than harmful functions. This indicates that other i-EVs components (proteins and/or RNAs) are mostly responsible for harmful astrocyte transformation. A1 astrocyte conversion likely occurred also in vivo in LPC-treated mice that received i-EVs for 4 days (Fig. 3a). Indeed, GFAP-positive astrocytes, present at myelin lesion, showed decreased PTX3 labelling and increased, albeit non significant, immunoreactivity for the A1 marker C3a compared to saline-injected mice (Fig. 7e, f) No major changes in A1 and A2 markers occurred at protein and mRNA levels in astrocytes exposed to MSC-EVs in vivo and in vitro (Fig. 7c–f).

Three microglial mediators have been shown to be both necessary and sufficient for A1 astrocyte conversion, i.e. IL-1a, C1q and TNFα. Of them, TNFα and IL1-a are mostly released by immune cells in EV-associated form [29, 86], while both TNFα and C1q were previously detected in microglia-derived EVs [17, 25, 98]. To assess the involvement of IL-1a, C1q and TNFα in A1 astrocyte transformation, cytokine profile of i-EVs was assessed with specific ELISA. Detectable amounts of IL-1a, C1q and TNFα were found in i-EVs isolated from 15 × 10^6^ microglial cells (IL1-a ~ 90 pg; C1q content ~ 78 pg; TNF-α content ~ 613 pg), supporting their role in harmful astrocyte transformation. Among the three inflammatory mediators, only TNF-α was suppressed in parental microglia by MSC conditioning at both protein and mRNA levels, as determined by ELISA (Fig. 7g) and qPCR (Fig. 2b). We, therefore, measured the TNFα content in i-EVs and MSC-EVs. i-EVs displayed about threefold higher levels of TNF-α compared to MSC-EVs (i-EVs TNF-α content ~ 613 pg; MSC-EVs TNF-α content ~ 168 pg), suggesting that the capacity of MSC-EVs to promote myelin repair may occur, at least in part, via TNF-α suppression. To assess TNF-α involvement in astrocyte activation, we pre-treated i-EVs with Etanercept (ETN, 200 ng ml^−1^), a TNF-α inhibitor, before co-exposing astrocytes to i-EVs and ETN. qPCR analysis showed that TNF-α inactivation prevented serping-1 upregulation and induced PTX3 expression in vitro, suggesting a shift of cultured astrocytes towards beneficial function (Fig. 7c, d). Immunolabelling for C3 in vivo revealed a significant decrease in the density of astrocytes positive for the A1 marker at lesions co-injected with i-EVs and ETN compared to i-EVs alone. However, PTX3 staining showed that the inhibitor caused by itself a decrease in the density of PTX3 positive astrocytes, being not able to prevent the decrease in PTX3^+^ astrocytes induced by i-EVs (Fig. 7e, f). Taken together, these data indicate that TNF-α is essential for the astrocyte response, despite its inactivation is not sufficient to prevent harmful astrocyte transformation in vivo.

### EVs binds to the OPC cell surface

**Fig. 8:**
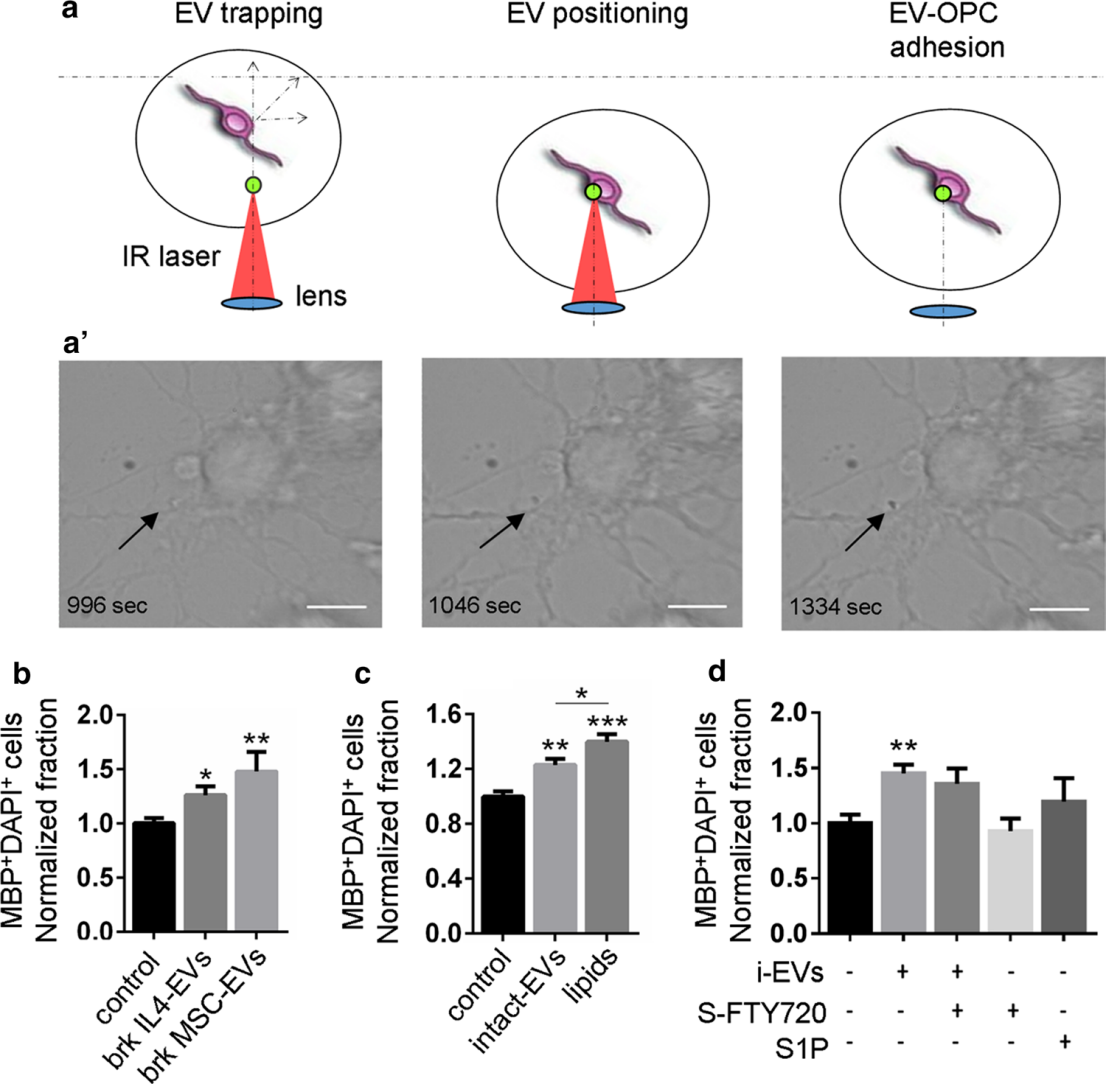
EVs efficiently interact with OPCs. **a** Schematic representation of EV delivery to OPCs by optical tweezers. EVs are first trapped above the OPCs by the IR laser tweezers (left), then the stage is moved in plane (*XY*) and the objective/trap is moved axially (*Z*) to set the EVs in contact with the OPCs (middle). The trapping laser is switched off to check whether EV adheres to the neuron membrane (right). **a′** Sequence of phase-contrast images showing one example of EV driven to an OPC following the procedure described in **a** (scale bar 10 μm). **b**, **c** OPCs were maintained in control condition or exposed to intact EVs, broken EVs or the lipid extract of EVs for 2 days, fixed and stained for MBP. Histograms show the percentage of mature MBP^+^ oligodendrocytes in cultures exposed to broken EVs derived from IL-4 microglia (Brk IL4-EVs) or MSC-treated cells (Brk MSC-EVs) (**b**; *n* = 3; Kruskal–Wallis test *p* = 0.0049 with Dunn’s multiple comparisons test versus control), intact EVs or native lipids (lipids) extracted from IL4-EVs (**c**; one-way ANOVA *p* = 0.0001 with Holm-Sidak’s multiple comparisons test). Data have been normalized to control. **d** Percentage of differentiated MBP^+^ oligodendrocytes in cultures exposed for 2 days to i-EVs alone or in combination with S-FTY720-Vinylphosphonate, FTY720-Vinylphosphonate alone or S1P. Data have been normalized to control (*n* = 3; Kruskal–Wallis test *p* = 0.0009 with Dunn’s multiple comparisons test versus control)

While mechanisms underlying astrocyte reaction to microglial EVs have been previously studied [25, 95], how EVs influence OPC activity is completely unknown. We explored whether EVs physically interact with OPCs. We placed single EV in contact with cultured OPCs using optical tweezers and monitored EV-OPC dynamics using time lapse microscopy (Fig. 8a). We observed that about 70% of NS-EVs adhered to the OPC surface (17 out of 24), suggesting that, in principle, most EVs could influence OPC function.

### The pro-differentiation activity of EVs depends on the lipid components of EVs

To investigate whether direct effects of EVs were mediated by factor(s) present at the surface or in the lumen of EVs, IL-4-EVs or MSC-EVs were busted by hypo-osmotic shock [34] and membrane fragments pelleted to remove soluble components were added to cultured OPCs. Broken EVs, depleted of their content, retained the capability to promote OPC maturation (Fig. 8d), revealing that surface component(s) of EVs were mainly driving this process. To distinguish between protein and lipid component(s), we exposed OPCs to the lipid fraction of IL4-EVs (Fig. 8e) or i-EVs (not shown). EV lipids enhanced OPC maturation even more efficiently than intact EVs (Fig. 8e), indicating that the pro-differentiating activity of EVs is mostly attributable to their lipid components. Finally, given that previous studies have shown that S1P and its analog FTY720-p promote OPC differentiation and myelin deposition [23, 65], we asked whether vesicular S1P may be implicated in the pro-differentiating activity of the EVs. We treated OPCs with the S1P receptor antagonist S-FTY720-Vinylphosphonate and found no changes in EV-induced OPC differentiation to MBP^+^ oligodendrocytes (Fig. 8f), thus ruling out a role of vesicular S1P in OPC maturation.

## Discussion

Our study reveals a previously unrecognised role of EVs produced by microglia in the control of remyelination, the spontaneous process by which OPCs differentiate into myelin-forming cells, restore myelin sheaths to protect axons from degeneration and allow fast signal transmission [24]. Specifically, we show that exogenous EVs from differentially conditioned microglia exert opposite effects on remyelination, with either pro-regenerative or detrimental actions, depending on the type of conditioning stimulus. Importantly, our study also unveils an unprecedented role for astrocytes in mediating the detrimental effects of EVs from pro-inflammatory microglia. Finally, it gives a first identification of the distinct molecular components of EVs involved in astrocyte detrimental transition as well as in promotion of OPC migration and/or differentiation, thus unveiling new targets for modulation of myelin repair.

### EVs mediate microglial action on oligodendrocytes

Previous evidence had shown that microglial phenotype strongly influences remyelination [30, 72] and that a predominant pro-regenerative response of microglia is required for efficient myelin repair after damage [64]. However, the mode(s) of action of microglia in fostering or inhibiting myelin repair was largely unclear. In the present study, by utilising a combined approach (immunohistochemistry and EM), we unequivocally show that EVs released by pro-regenerative microglia promote OPC recruitment and differentiation at LPC-induced myelin lesions, while i-EVs, derived from inflammatory microglia, block remyelination. Our study advances current knowledge by showing that (1) dark, reactive microglia are recruited at myelin lesion and release EVs, as indicated by EM, and (2) EVs are sufficient to mediate either detrimental or beneficial function on myelin forming cells, reflecting the phenotype of donor microglia, upon exogenous administration to the lesion site. EVs remain intact for a short time at the injection site, likely as a consequence of lysosomal degradation [15] or cell fusion and cargo dilution [75], but cause a prolonged modulation of oligodendrocytes surrounding the lesion. This is in line with a recent study showing that microglial EVs engineered to deliver an anti-inflammatory cytokine IL-4 induce a long-lasting immune modulation in the EAE model of multiple sclerosis [15]. However, whether EVs with opposite action on OPCs may be generated endogenously by microglia and may be relevant for the course of de- and re-myelination in animal models of MS still remains unclear. Selective tools to manipulate endogenous EV production are needed to overcome this limitation of our study and to analyse the role of microglial EVs in a more physiological setting.

### A1 astrocytes mediate detrimental action of i-EVs on OPCs

The major finding of this paper is the demonstration that astrocytes mediate the detrimental action of inflammatory microglia-derived EVs on OPCs. While i-EVs by themselves favor differentiation of OPCs in monoculture, they cause a clear block of OPC maturation when OPCs are cocultured with astrocytes, thereby mimicking the inhibitory action of i-EVs at myelin lesion in the in vivo LPC-demyelination model. Importantly, inhibition of OPC maturation in the OPC/astrocytic co-cultures is likely paralleled by conversion of astrocytes into A1 harmful cells. Indeed, our previous work revealed that cultured astrocytes become hypertrophic and upregulate inflammatory markers when exposed to i-EVs [95]. By analysing the expression and immunoreactivity of a few specific A1 and A2 markers in this study, we suggest that astrocytes acquire a harmful A1 phenotype both in vitro and in the LPC-mouse model of myelin lesion. A1 astrocytes were recently proven to slow OPC differentiation and to damage differentiated oligodendrocytes [20, 99] and have been observed in demyelinating plaques of MS patients [55]. By showing that microglial i-EVs cause A1 astrocyte transformation, we add an important piece of information towards the understanding of the mechanism possibly responsible for remyelination failure in MS. We hypothesise that demyelinating lesions may fail to remyelinate because EVs produced by chronically activated microglia block OPC differentiation by inducing harmful astrocyte conversion, thus nullifying the direct pro-myelinating action of EVs. In line with our study, previous works highlighted the detrimental effect of chronically activated astrocytes on myelin repair, by showing that depletion of reactive astrocytes at the chronic phase of EAE improves EAE clinical outcome [62] and that remyelination by transplanted OPCs is less extensive in areas of demyelination that contain astrocytes than in astrocyte-free areas [8]. A few proteins released by astrocytes, including CXCL10 [67] and endothelin-1 [42], inhibit OPC maturation and may be involved in remyelination failure. By contrast, no molecules with the capacity to damage OPCs/oligodendrocytes have been identified so far in the secretome of inflammatory microglia, which rather contains protective agents for myelin-forming cells [24, 63].

### EV signals driving astrocytes towards oligotoxic cells

Although our analysis indicates that EVs carry the glycolipid lactosylceramide (LacCer) and S1P, known inducers of pro-inflammatory astroglial activation in EAE [19, 62, 81], the lipid extract of i-EVs does not drive harmful transformation in cultured astrocytes, implicating the protein and/or RNA cargoes of i-EVs in the acquisition of detrimental astroglial function. Previous studies reported that activated microglia release three mediators that are necessary and sufficient for astrocyte conversion to harmful cells, i.e. TNF-α, IL-1a and C1q [55]. Here we show that (1) i-EVs carry the three inducers of A1 astrocytes, (2) TNF-α is decreased in MSC-EVs, which do not cause harmful astrocyte transformation, and (3) TNF-α inactivation partially inhibits the proinflammatory astrocyte response. Besides identifying TNF-α as a target of MSC action in microglia, this evidence implicates vesicular TNF-α in the astrocyte shift towards oligotoxic cells. However, other molecules may be sorted in MSC-EVs, such as TGF-β or FGF [2], which may counteract the action of the inflammatory cargoes, preventing A1 astrocyte activation.

Elevated production of TNF-α was previously reported in the serum, cerebrospinal fluid and brain plaques of MS patients [59], correlating with disease severity or exacerbation [58, 93]. In addition, it was recently shown that TNF-α is mostly produced by microglia and macrophages in acute and progressive EAE, enhancing clinical disability [91]. Whether the content of TNF-α is elevated in microglial EVs of MS patients, especially those with progressive MS, is an important question for future studies that may lead to the identification of novel disease biomarkers. In devising possible therapeutic strategies to promote myelin repair, it should be noted, however, that TNF-α has been attributed both detrimental and protective roles in MS. Specifically, the membrane bound form of the cytokine was shown to sustain reparative processes via activation of type 2 TNF-α receptor (TNFR2) in oligodendrocytes [13], preventing the clinical use of TNF-α blockers in MS [47].

### Key role of lipids in the pro-myelinating action of EVs

Another key observation of our study is that the lipid content of all types of microglial EVs has a direct impact on OPCs, directing both their recruitment and differentiation.

Through measurement of sphingolipid content and the use of a potent S1P receptor antagonist S-ene-FTY720 Vinylphosphonate, we show that S1P is secreted by microglia in association with EVs and acts as an attractive guidance cue for OPCs. Although the recent use of the S1P analog fingolimod/FTY720, which activates S1P receptors, to treat MS has fueled research on the direct effects of S1P on oligodendrocytes [38], to our knowledge, our data provide the first demonstration of a central role for EVs-associated S1P in OPC migration, the first key step in myelin repair. Other chemotactic molecules, such as Wnt3a [45] and the endocannabinoid anandamide [34] are packaged in microglial EVs, revealing the important role of EVs as vehicles of chemotactic signals [52]. Through EV fractionation, we also show that lipids of all EV types drive OPC maturation, making lipid research a hotspot in the field of myelin repair. We rule out the involvement of S1P in the pro-differentiating action of EVs, since the capacity of EVs to accelerate OPC maturation was unchanged under pharmacological blockade of S1P receptors. We anticipate that identification of lipids responsible for the pro-differentiating action of microglial EVs may help to design new therapeutics to foster myelin repair.

Previous studies implicated the miRNA cargo of EVs in myelin repair, rather than the lipid content. Pro-myelinating exosomes derived by peripheral immune cells were previously identified in rat serum [76, 77]. Their pro-differentiating activity was partially ascribed to the delivery of miR-219 to OPCs, a microRNA that regulates multiple genes in the differentiation pathway [76]. Although miR-219 is present in EVs derived from microglia and is upregulated in i-EVs [75], our biochemical experiments indicate that the lipid fraction of EVs, which is free of nucleic acids, retains the ability to drive OPC maturation, arguing against a major involvement of miRNAs in the direct pro-differentiating action of microglial EVs. Nevertheless, the RNA and/or protein cargoes of EVs may indirectly regulate OPC maturation by influencing the microenvironment around the lesion.

### The unique pro-myelinating activity of EVs derived from microglia co-cultured with MSC

We show that MSC-EVs have a unique capacity to stimulate the endogenous reparative response of OPCs at myelin lesion. The pro-myelinating action of MSC-EVs exceeds that of IL4-EVs both in vitro and in vivo. This suggests that well-known effects of MSCs in EAE rely, at least in part, on the paracrine effect of MSCs on microglia phenotype and microglia communication with OPCs and astrocytes during remyelination. Further work remains to be done to define the minimal components of MSC-EVs required to drive efficient remyelination. Selected luminal components of MSC-EVs (proteins and/or RNAs) in combination with pro-differentiating lipid(s) may be assembled in EV mimetics of low complexity and with low chance of off-target effects, which may be ideal tools to develop novel therapeutic approaches in chronic MS.

## Electronic supplementary material

Below is the link to the electronic supplementary material.

Supplementary material 1 (PDF 867 kb)

## Abbreviations

CC: Corpus callosum
cryo-EM: Cryo-electron microscopy
dpl: Days post lesion
DTI: Diffusion tensor imaging
DRG: Dorsal root ganglion
ETN: Etarnecept
EVs: Extracellular vesicles
i-EVs: EVs from inflammatory microglia
IL4-EVs: EVs derived from IL4-treated microglia
MSC-EVs: EVs derived from MSC-treated microglia
ICC: Immunofluorescence
IHC: Immunohistochemistry
i-microglia: Inflammatory microglia
LPC: Lysolecithin
MSCs: Mesenchymal stem cells
MS: Multiple sclerosis
OPCs: Oligodendrocyte precursor cells
MACS: Magnetic-activated cell sorting
MRI: Magnetic resonance imaging
WB: Western-blot
