# Supplementary material for: Detrimental and protective action of microglial extracellular vesicles on myelin lesions: astrocyte involvement in remyelination failure
Source: Acta Neuropathol. 2019 Jul 30;138(6):987–1012. doi: 10.1007/s00401-019-02049-1 (PMC6851224; doi:10.1007/s00401-019-02049-1)
Supplement: Supplementary file 1 — Supplementary material 1 (PDF 867 kb) [file 401_2019_2049_MOESM1_ESM.pdf]

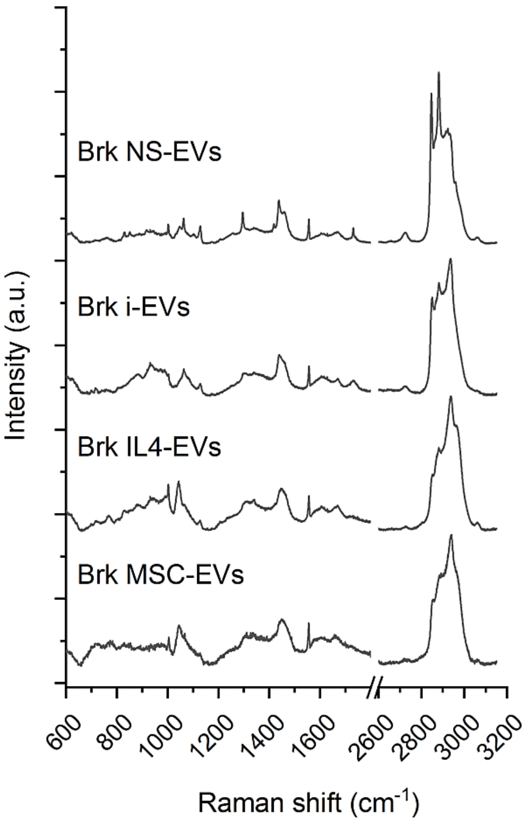

Supplementary Figure 1: Raman spectra of broken EVs. Mean Raman spectra obtained using 532 nm laser line from broken (Brk) EVs of NS or polarized microglia. All spectra were baseline corrected, aligned and normalised before averaging.

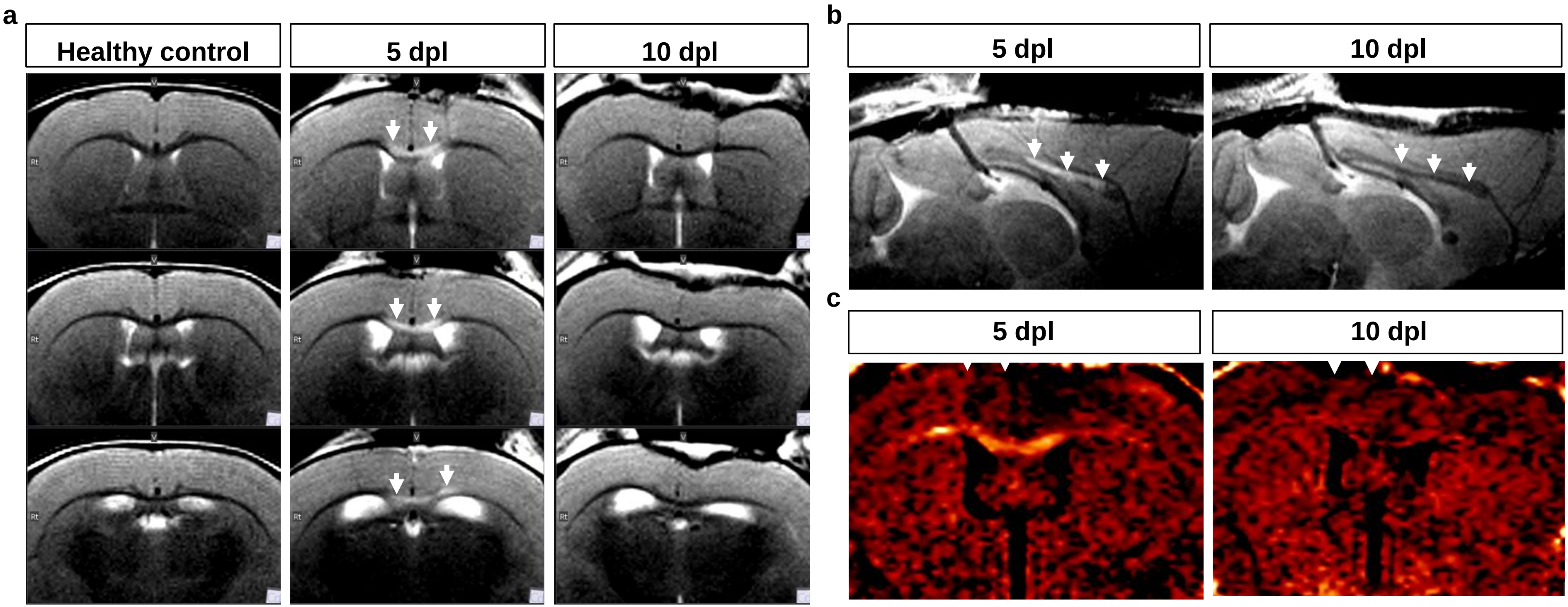

Supplementary Figure 2: MRI analysis of the corpus callosum (CC) of LPC-treated old mice at different time points.
At 5 dpl, lesions along the CC are clearly observed as hypersignal on T2 weighted images (arrows on **a**, coronal sections; and **b**, sagittal sections). At 10 dpl, CC signal returned to normal, almost similar to healthy controls. CC demylination was also observed with the increase of water molecule diffusivity perpendicular to fibers at 5 dpl (**c**, left image) which was decreased to normal at 10 dpl (**c**: right image).

Suppl Fig. 3

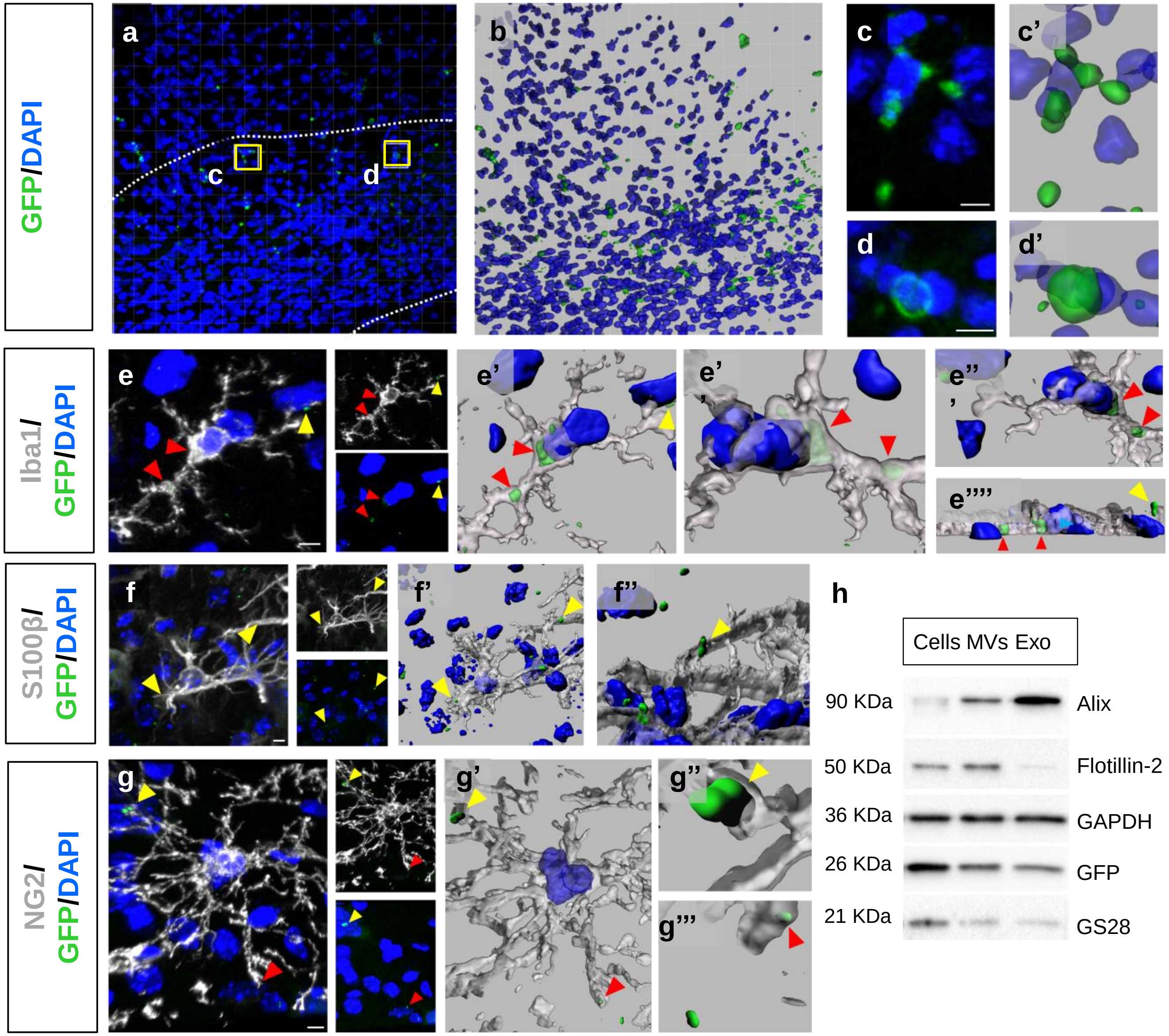

Supplementary Figure 3: GFP-labelled EVs at the lesion site. a Photomichrograph showing GFP⁺ EVs at the lesion site (dotted line) 20 minutes after injection. **b** Imaris 3D reconstruction of the field of view shown in a. **c-d** High-magnification of the insets in a. **c'-d'** Imaris 3D reconstruction of the field of view in c and d. **e-g** Co-staining of GFP-labelled EVs (green) with Iba1⁺ microglia (grey, in e), S100β⁺ (grey, in f) or NG2⁺ OPCs (grey, g)**.** Insets on the right are co-staining of GFP⁺ EVs and the lineage marker (top inset) or dapi (bottom) to reveal the internalized EVs (red arrowheads). Yellow arrowheads indicate GFP+EVs outside the cell or anchored to the membrane. **Inset e'-g'** Imaris 3D reconstruction of correspondent confocal images. **e''-e'''', f'' g''-g'''** High-magnification of Imaris 3D reconstruction. Rotations and zoom have been applied to better visualize the EVs and their relations with the cells. Scale bars: **a** 10µm and **c-g** 5µm. **h** Western blot analysis of MVs- and exosomes-enriched fractions produced from rat microglia expressing cytosolic GFP with the indicated antibodies. Note the presence of GFP in both types of EVs. MVs and exosomes purity is indicated by low immunoreactivity for the Golgi marker GS28. The first lane shows 5 µg of cell lysates.

Suppl. Fig. 4

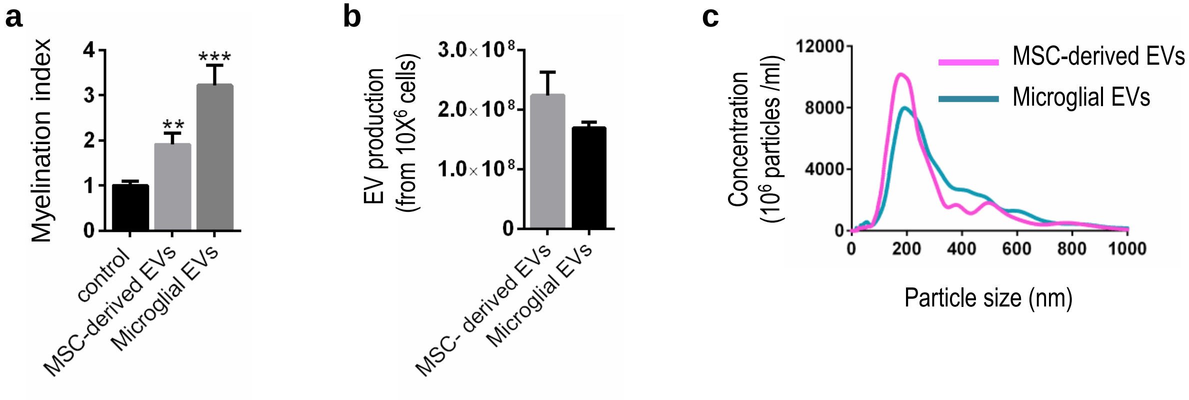

Supplementary Figure 4: EVs produced by MSCs are less effective than EVs produced by MSC-treated microglia in promoting myelination *in vitro*.

a Histograms show myelination index in OPC-DRG co-cultures exposed to EVs released by MSCs (MSC-derived-EVs) or MSC-treated inflammatory microglia (microglial EVs). (number of experiments (n)= 3, Kruskal-Wallis test p< 0,0001 with Dunn's multiple comparison). **b** Histograms show production of EVs from $1x10^6$ MSCs (MSC-derived-EVs) or MSC-treated inflammatory microglia (Microglial EVs) during ATP stimulation for 30 min. Mann Whitney test p=0,6000. **c** Size profile of MVs-enriched fraction, pelleted from $1x10^6$ MSCs or MSCs-treated microglia. (MSC-derived MVs: Mean 289.45±158.55, Mode 192.25; Microglial EVs: Mean 330.53± 162.67, Mode 228.60; n=3).
